# METTL14 downregulation drives S100A4^+^ monocyte-derived macrophages via MyD88/NF-κB pathway to promote MAFLD progression

**DOI:** 10.1038/s41392-024-01797-1

**Published:** 2024-04-17

**Authors:** Yue-fan Wang, Wen-li Zhang, Zhi-xuan Li, Yue Liu, Jian Tan, Hao-zan Yin, Zhi-chao Zhang, Xian-jie Piao, Min-hao Ruan, Zhi-hui Dai, Si-jie Wang, Chen-yang Mu, Ji-hang Yuan, Shu-han Sun, Hui Liu, Fu Yang

**Affiliations:** 1 The Third Department of Hepatic Surgery, Eastern Hepatobiliary Surgery Hospital Affiliated to Naval Medical University, 200438 Shanghai, China; 2 The Department of Medical Genetics, Naval Medical University, 200433 Shanghai, China; 3 Translational Medicine Research Center, Medical Innovation Research Division and Fourth Medical Center of the Chinese PLA General Hospital, 100048 Beijing, China; 4 The Department of Pharmaceutical Analysis, School of Pharmacy, Naval Medical University, 200433 Shanghai, China; 5 Key Laboratory of Biosafety Defense, Ministry of Education, 200433 Shanghai, China; 6 Shanghai Key Laboratory of Medical Biodefense, 200433 Shanghai, China

**Keywords:** Epigenetics

## Abstract

Without intervention, a considerable proportion of patients with metabolism‐associated fatty liver disease (MAFLD) will progress from simple steatosis to metabolism‐associated steatohepatitis (MASH), liver fibrosis, and even hepatocellular carcinoma. However, the molecular mechanisms that control progressive MAFLD have yet to be fully determined. Here, we unraveled that the expression of the N6-methyladenosine (m6A) methyltransferase METTL14 is remarkably downregulated in the livers of both patients and several murine models of MAFLD, whereas hepatocyte-specific depletion of this methyltransferase aggravated lipid accumulation, liver injury, and fibrosis. Conversely, hepatic *Mettl14* overexpression alleviated the above pathophysiological changes in mice fed on a high-fat diet (HFD). Notably, in vivo and in vitro mechanistic studies indicated that METTL14 downregulation decreased the level of GLS2 by affecting the translation efficiency mediated by YTHDF1 in an m6A-depedent manner, which might help to form an oxidative stress microenvironment and accordingly recruit *Cx3cr1*^+^*Ccr2*^+^ monocyte-derived macrophages (Mo-macs). In detail, *Cx3cr1*^+^*Ccr2*^+^ Mo-macs can be categorized into M1-like macrophages and S100A4-positive macrophages and then further activate hepatic stellate cells (HSCs) to promote liver fibrosis. Further experiments revealed that CX3CR1 can activate the transcription of S100A4 via CX3CR1/MyD88/NF-κB signaling pathway in *Cx3cr1*^+^*Ccr2*^+^ Mo-macs. Restoration of METTL14 or GLS2, or interfering with this signal transduction pathway such as inhibiting MyD88 could ameliorate liver injuries and fibrosis. Taken together, these findings indicate potential therapies for the treatment of MAFLD progression.

## Introduction

Metabolism‐associated fatty liver disease (MAFLD), formerly known as nonalcoholic fatty liver disease, has emerged as the prevailing chronic liver disease globally in the twenty-first century due to changes in lifestyle and the increased prevalence of obesity, both of which are intimately associated with metabolic dysfunction.^1,2^ MAFLD includes a range of disease conditions from simple steatosis to metabolism-associated steatohepatitis (MASH), liver fibrosis, cirrhosis, and hepatocellular carcinoma (HCC). However, there are no particular medications to treat MASH. Therefore, further research is required to comprehend the pathogenesis and progression of MAFLD and to create novel therapeutic strategies.

Immune activation is a key factor in the occurrence and progression of MAFLD.^3^ Previous studies have shown that intrahepatic macrophages can be activated by lipid accumulation in the liver cells of MASH patients to become proinflammatory cells.^2^ However, intrahepatic macrophages are a heterogeneous and dynamic population, and it is unclear which intrahepatic macrophage subsets are involved in the initiation and development of MAFLD. In the healthy state, Kupffer cells (KCs), are the predominant population, self-renewing and interacting with hepatocytes and parenchymal cells. During liver injury, such as MASH, KCs are depleted, and intrahepatic bone marrow monocyte-derived macrophages (Mo-macs) can be recruited from circulating monocytes.^4^ However, researchers are still uncertain about the recruitment mechanism, formation, and characteristics of Mo-macs, as well as their roles in MAFLD progression to date.

Recent studies indicate that there is an observed disorder in N6-methyladenosine (m6A) in MAFLD mouse models and liver cells treated with free fatty acids (FFAs), while aberrant m6A modification is ascribed to the upregulation of the m6A “writer” protein METTL3, which is induced by hepatic lipotoxicity. Knockdown of METTL3 could promote liver autophagy and lipid drop clearance. Overexpression of METTL3 could inhibit these processes.^5^ Specific knockout of METTL3 in myeloid cells prevented age-related and diet-induced MAFLD progression.^6^ These existing studies indicate that RNA m6A modification system is disoriented in the progression of MAFLD and participates in the progression of the disease. However, there is little understanding of the role and clinical importance of RNA m6A alteration in the progression of steatosis-MASH-liver fibrosis at present.

In our previous work, we found that the METTL14-regulated m6A modification could enhance the recognition of pri-miRNA 126 by DGCR8 and promote the production of mature miR-126.^7^ In later work, we found that the expression level of METTL14 in liver tissues of MAFLD mice was much lower than that of control mice. In addition to this, it was shown that the expression of METTL14 in human liver tissues diagnosed with MAFLD was comparatively lower than in normal liver tissues. Therefore, we speculated that METTL14 may play a regulatory role in the progression of MAFLD.

## Results

### METTL14 downregulation in human and murine MAFLD

**Fig. 1:**
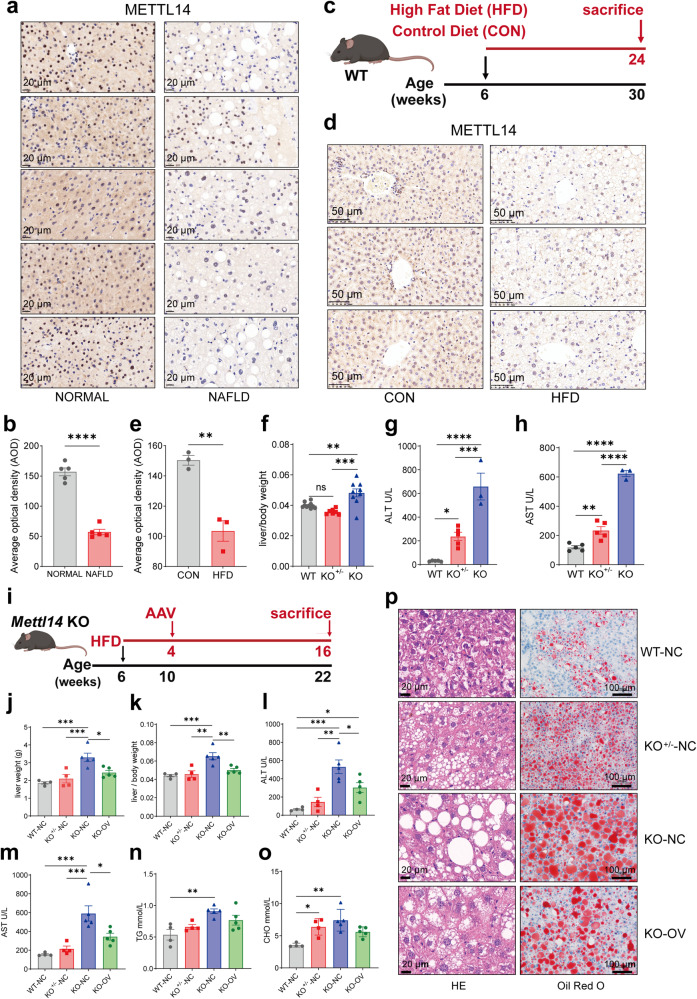
METTL14 is downregulated in the livers of mice with HFD and in human MAFLD. **a** Sections of human healthy liver (*n* = 5) and metabolic fat liver disease (MAFLD) (*n* = 5) were analyzed by immunohistochemical analysis for METTL14. Representative images are shown. **b** Relative quantitative analysis of METTL14 in normal human liver and metabolic fat liver tissues (*n* = 5). **c** Schematic diagram of the dietary feeding scheme. Six-week-old C57BL/6 wild-type mice were fed either a control diet (CON) or a 60% high-fat diet (HFD) for 24 weeks. **d** Mice were sacrificed, and liver sections were analyzed by immunohistochemical analysis for METTL14 expression (*n* = 3). **e** Relative quantitative analysis of METTL14 in liver tissues of mice fed the HFD and control diet (*n* = 3). **f** Comparison of the liver-to-body weight ratio of 8-week-old wild-type (WT, *n* = 10), heterozygous knockout mice (KO^+/−^, *n* = 7) and homozygous knockout mice (KO, *n* = 9). **g**, **h** Serum ALT (**g**) and AST (**h**) in wild-type (WT, *n* = 5), heterozygous knockout mice (KO^+/−^, *n* = 5) and homozygous knockout mice (KO, *n* = 3). **i** Schematic diagram of 6-week-old WT, KO^+/−^ and KO mice fed a HFD for 16 weeks. KO mice fed a HFD for 4 weeks were randomly injected with AAV-8 overexpressing METTL14 (AAV-OV) or control vector (AAV-NC) through the caudal vein, while WT mice and KO^+/−^ mice fed a HFD for 4 weeks were injected with AAV-NC. **j**–**o** Comparison of liver weight (**j**) and liver to body ratio (**k**), serum ALT (**l**) and AST (**m**), serum triglycerides (TG, **n**) and cholesterol (CHO, **o**) of WT (*n* = 4), KO^+/−^ (*n* = 4), KO injected with empty AAV-8 vector (KO-NC, *n* = 5) and KO injected with AAV-8 vector overexpressing METTL14 (KO-OV, *n* = 5). **p** H&E (left) and Oil Red O (right) staining of liver sections from HFD-treated WT (*n* = 4), KO^+/−^ (*n* = 4), KO-NC (*n* = 5) and KO-OV (*n* = 5) mice are shown. Data are represented as mean ± SEM. NS not significant, *p < 0.05, **p < 0.01, ***p < 0.001, ****p < 0.0001. The mouse image was created with BioRender.com

To assess METTL14 expression in MAFLD progression, we performed immunohistochemical analyses in tissue samples from patients with healthy liver and MAFLD. These studies revealed a robust downregulation of METTL14 in MAFLD liver tissue, characterized by macrovesicular steatosis (Fig. 1a, b). Next, to determine the clinical relevance of METTL14 in the pathogenesis of MAFLD, we investigated whether there were histological and transcriptomic differences between different METTL14 levels in the liver with the data from the GTEx project.^8^ We established groups of patients with high and low METTL14 expression using median METTL14 levels in 161 individuals. Patients with mild, moderate, or severe steatosis were more observed in the low-METTL14 group (Supplementary Fig. 1a, b). METTL14 expression was lowered in the group of patients with mild, moderate, or severe steatosis compared with patients with minimal or no steatosis (Supplementary Fig. 1c, d). Prompted by these findings, we expanded our analysis of METTL14 expression in multiple murine MAFLD models. First, we fed mice with a 60% high-fat diet (HFD) for 24 weeks (Fig. 1c), which is an excellent tool for studying the incremental steps of MAFLD progression.^9^ In line with previous reports, HFD-treated mice developed hepatic steatosis with ballooned hepatocytes. Immunohistochemical analysis confirmed a strong downregulation of METTL14 in the livers of MAFLD mice (Fig. 1d, e and Supplementary Fig. 1e), which is consistent with our findings in human MAFLD. We then generated another two diet-induced MAFLD models, including mice fed with a Western diet (WD) and an Amylin-Liver Nash (AMLN) diet (Supplementary Fig. 1f–h and Supplementary Fig. 1i–k).^10,11^ Interestingly, METTL14 expression was similarly decreased in the livers of WD-fed and AMLN-fed mice compared to their respective controls (Supplementary Fig. 1h, k).

To study whether downregulation of METTL14 was indeed causally involved in the initiation and progression of MAFLD, we generated genetically modified mice with hepatocyte-specific depletion (Albumin-Cre) of METTL14 (heterozygous knockout: KO^+/−^; homozygous knockout: KO; wild type: WT). We generated a *Mettl14* loxP floxed allele (Supplementary Fig. 2a, b) and verified the reduction of *Mettl14* mRNA and protein abundance (Supplementary Fig. 2c–f). The liver weight and body weight of KO mice were markedly reduced at 3 months compared to those of KO^+/−^ and WT mice (Supplementary Fig. 2g, h); however, the liver to body weight ratio increased (Fig. 1f). Histology assessments revealed hepatocyte swelling, disordered structures of hepatic plates as well as infiltration of lipid droplets and inflammatory cells in the livers of KO mice (Supplementary Fig. 2i). These results indicated that METTL14 played a vital role in body growth and liver tissue development to some extent. Additionally, ALT and AST levels were significantly increased in KO^+/−^ and KO mice at 3 months, and the upregulation of ALT and AST levels in KO mice was much more obvious (Fig. 1g, h), revealing that hepatocyte-specific depletion of METTL14 leads to severe liver injuries.

Prompted by these findings, we utilized 6-week-old WT and *Mettl14* knockout (KO^+/−^, KO) mice under HFD treatment for 16 weeks to further study the role of METTL14 in mediating MAFLD. WT and KO^+/−^ mice were treated with adeno-associated virus-8 control vector (AAV-NC) via tail vein injection after four weeks of feeding, while KO mice were randomly injected with AAV-NC or AAV overexpressing METTL14 (AAV-OV) through the caudal vein and then fed a HFD for another 12 weeks (Fig. 1i). Compared with WT or KO^+/−^ mice injected with AAV-NC (WT-NC or KO^+/−^-NC), KO mice injected with AAV-NC (KO-NC) were significantly aggravated from 16-week HFD-induced hepatic steatosis, as shown by increased liver weights, the ratio of liver to body weight, ALT, AST and blood lipid levels, such as triglyceride (TG) and cholesterol (CHO) (Fig. 1j–o). As expected, treatment of KO-NC mice with HFD resulted in more progressive steatosis, ballooning, and pronounced lobular inflammation compared with those of WT-NC and KO^+/−^-NC mice, which was also verified by MASH Activity Score (MAS) assessment and Oil red O staining (Fig. 1p and Supplementary Fig. 2j, k). In contrast, KO mice administered AAV-OV (KO-OV, AAV overexpressing METTL14) exhibited lower liver weight, the liver-to-body weight ratio, ALT and AST levels than the KO-NC group (Fig. 1j–m), as well as lower lipid infiltration in histological analysis of livers (Fig. 1p and Supplementary Fig. 2j, k), revealing that METTL14 restoration reduced lipid accumulation and abolished the progression of MAFLD. Collectively, these findings substantiated the causal role of METTL14 in the progression of MAFLD and hinted at potential therapeutic strategies.

### GLS2 was downregulated in both KO mice and MAFLD mice and consequently promoted the oxidative stress microenvironment

**Fig. 2:**
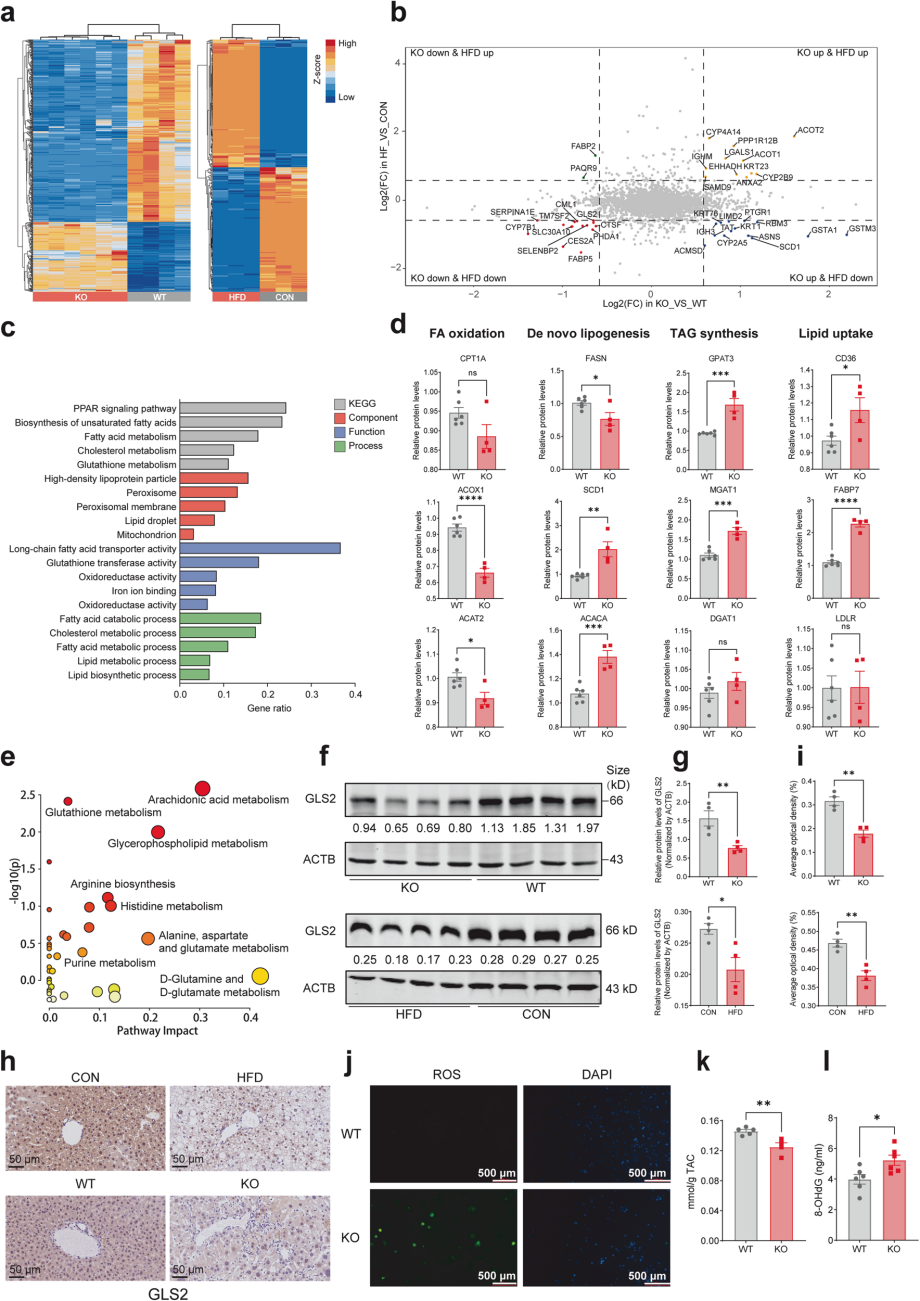
GLS2 was downregulated in both KO mice and HFD mice and consequently promoted the oxidative stress microenvironment. **a** Heat maps presenting differentially expressed proteins (DEPs) in HFD vs control diet-fed mice (*n* = 3) and KO vs WT mice (*n*_KO_= 4, *n*_WT_ = 6), respectively. **b** Nine quadrant diagrams presenting the overlapping upregulated proteins and/or downregulated proteins among HFD and KO mice from proteomics sequencing (the dashed lines indicating the fold changes are at 1.5). **c** GO and KEGG analyses of differentially expressed proteins indicating lipid metabolic alterations among KO and WT mice. **d** The levels of specific markers related to lipid metabolism including fatty acid oxidation (CPT1A, ACOX1, ACAT2), de novo lipogenesis (FASN, SCD1, ACACA), TAG synthesis (GPAT3, DGAT1, MGAT1) and lipid uptake (CD36, FABP7, LDLR). **e** Overview of the pathway analysis based on metabolite alterations in KO mice from metabolomics analysis. **f** Western blot indicating the levels of GLS2 in 8-week-old WT and KO mice (top) and HFD-fed and control diet-fed mice (bottom). Representative images are shown (*n* = 4). **g** Relative quantitative analysis showing the levels of GLS2 in WT and KO mice (top) and HFD and control diet mice (bottom). **h** Immunohistochemical staining analysis of GLS2 in WT and KO mice, HFD-fed mice and control diet-fed mice (*n* = 4, scar bar = 50 μm). **i** Relative quantitative analysis of GLS2 in WT and KO mice (top) and HFD-fed and control diet-fed mice (bottom) (*n* = 4 respectively). **j** DCFH-DA was used to display the levels of intracellular ROS in primary cultured hepatocytes isolated from WT and KO mice. Representative images are shown(scar bar = 500 μm). **k** TAC levels showing total antioxidant capacity using ABTS methods in KO (*n* = 4) and WT (*n* = 5) mice. **l** ELISA showing the levels of 8-OHdG in KO and WT mice (*n* = 6 respectively). Data are represented as mean ± SEM. NS not significant, *p < 0.05, **p < 0.01, ***p < 0.001, ****p < 0.0001

To obtain a comprehensive explanation for the roles of METTL14 in MAFLD progression, we performed proteomic analysis of liver tissues from the *Mettl14* KO (*n* = 6) and WT (*n* = 4) mice, as well as HFD-fed (*n* = 3) and control diet (CON)-fed (*n* = 3) mice (Fig. 2a and Supplementary Tables 1, 2), and then carried out differentially expressed protein (DEP) conjoint analysis among four groups of mice (Fig. 2b). Gene Ontology (GO) and Kyoto Encyclopaedia of Genes and Genomes (KEGG) analyses employing these DEPs firmly revealed that METTL14 deficiency significantly impacted fatty acid metabolism and biosynthesis process (Fig. 2c). Subsequently, we analyzed gene expression patterns associated with lipid homeostasis in the liver after METTL14 deficiency employing lipid metabolic markers such as FA oxidation markers (CPT1A, ACOX1, ACAT2), de novo lipogenesis markers (FASN, SCD1, ACACA), TAG synthesis markers (GPAT3, DGAT1, MGAT1), and lipid uptake markers (CD36, LDLR, FABP7). Depletion of METTL14 was discovered to inhibit fatty acid oxidation, increase lipid synthesis and uptake, and consequently hasten lipid accumulation (Fig. 2d). To further investigate the effects of METTL14 deficiency on the metabolic microenvironment in mice, we performed a full-spectrum metabolomics analysis of the liver tissues from KO and WT mice (Supplementary Table 3). It was discovered that D-glutamine and D-glutamate metabolism were most significantly altered in KO mice among the metabolic pathways (Fig. 2e). Glutamine metabolism is principally regulated by two enzymes: glutaminase (GLS) and glutamine synthetase (GS). Glutaminase isoforms are tissue-specific: kidney-type glutaminase (GLS1) and liver-type glutaminase (GLS2).^12^ The proteomic analysis revealed that GS was marginally elevated in the KO mice (FC = 1.37), whereas it remained relatively unchanged in the HFD-fed mice (FC = 0.95) (Supplementary Fig. 3a, b). In addition, the expression of GS did not alter remarkably in the patients ranging from no steatosis, minimal, mild, moderate to severe steatosis (Supplementary Fig. 3c, d). However, the proteomic analysis also revealed that GLS2 was downregulated in both KO mice and HFD-fed mice (Fig. 2b). The expression of GLS2 protein was additionally observed, revealing a significant downregulation of GLS2 in the liver tissues of HFD mice and KO mice as compared to their respective controls (Fig. 2f–i), which was also in line with previous studies.^13^ It was also found a substantial downregulation of GLS2 in mice fed with a Western diet (Supplementary Fig. 1h). Nevertheless, GLS2 exhibited a decreased trend in the AMLN group, but there was no significant statistical disparity (Supplementary Fig. 1k).

It has been reported that limited expression of GLS2 in hepatocytes causes a decrease in antioxidant capacity and an increase in reactive oxygen species (ROS) levels in cells.^14–16^ We subsequently assessed the antioxidant capacity and ROS levels in liver tissues of KO mice. Compared to WT mice, the level of ROS in the hepatocytes of KO mice increased (Fig. 2j), and the antioxidant capacity decreased substantially (Fig. 2k). We also conducted an analysis on the levels of 8-hydroxy-2 deoxyguanosine (8-OHdG), a widely recognized biomarker for assessing oxidative stress and DNA damage.^17^ As anticipated, our findings revealed an upregulation of 8-OHdG in the liver tissues of KO mice compared with WT mice (Fig. 2l).

To determine whether GLS2 downregulation in *Mettl14*-deficient mice led to liver injury and oxidative stress milieu, we constructed and validated an GLS2-upregulation plasmid vector (Supplementary Fig. 4a–c), then generated serotype 8 AAVs overexpressing GLS2 and performed an in vivo rescue assay on KO mice (Supplementary Fig. 4d). We first validated the overexpression of GLS2 in the liver tissues of mice overexpressing GLS2 (KO-OE) and mice expressing control vector (KO-CTL) (Supplementary Fig. 4e, f). Although the morphology of the liver and the liver to body weight ratio did not differ significantly between KO-OE and KO-CTL mice (Supplementary Fig. 4g, h), KO-OE mice had substantially lower serum ALT levels (Supplementary Fig. 4i). Rescued GLS2 expression remarkably reduced the ROS levels and promoted the antioxidant capacity in the KO mice (Supplementary Fig. 4j, k). More importantly, fibrotic markers (*Col1a1*, *Acta2*, and *Mmp2*) were significantly reduced in GLS2 upregulated KO mice (Supplementary Fig. 4l), as confirmed by Sirius red staining and Masson staining (Supplementary Fig. 4m). Furthermore, immunofluorescence analysis of α-SMA revealed lower activation of hepatic stellate cells (HSCs) in the KO-OE mice (Supplementary Fig. 4m), indicating that GLS2 restoration could alleviate liver injury and fibrosis in *Mettl14* deficient mice.

These results indicated that the reduced expression of METTL14/GLS2 protein might contribute to the development of an oxidative stress milieu, hence facilitating the progression of MAFLD.

### METTL14 regulates GLS2 expression in an m6A-dependent manner via YTHDF1

**Fig. 3:**
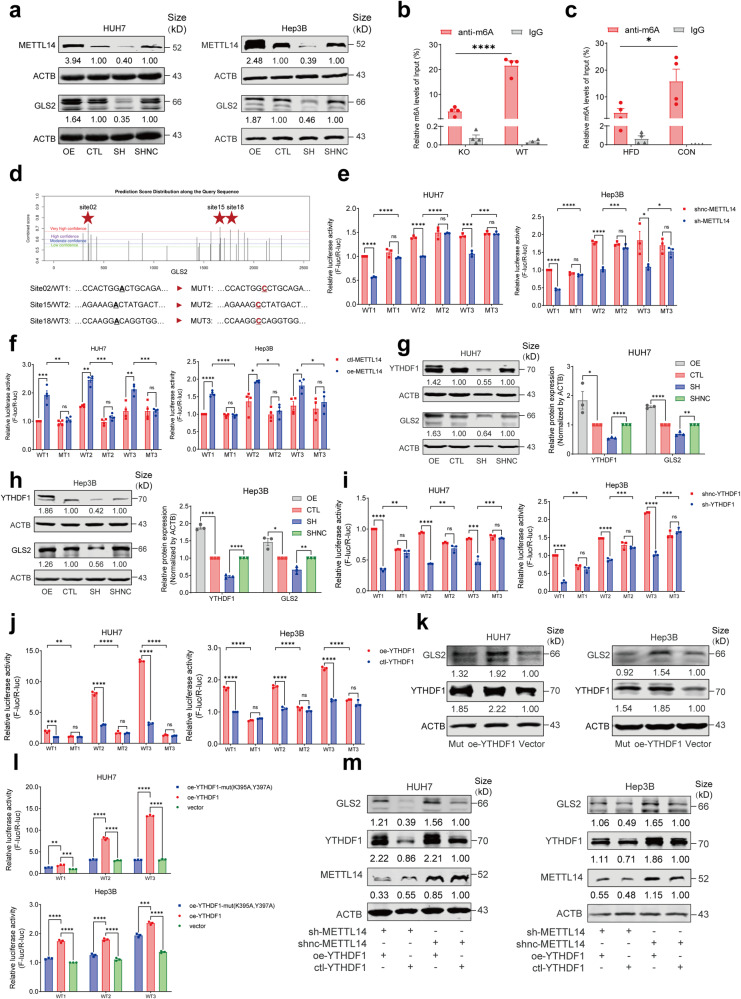
METTL14 regulates GLS2 expression in an m6A-dependent manner via YTHDF1. **a** Western blot showing METTL14 and GLS2 expression in HUH7 (left) and Hep3B (right) cells infected with METTL14 overexpression (OE) or shRNA lentivirus vector (SH). **b**, **c** Relative m6A enrichment in *Gls2* mRNA in liver tissues of WT and KO mice (**b**), HFD and control diet mice (**c**) by MeRIP-qPCR. *n* = 4. **d** The image showing the position and sequences of three potential m6A-binding sites with very high confidence of *GLS2* mRNA using online SRAMP database (https://www.cuilab.cn/sramp). **e** Relative luciferase activity of three mutant plasmids and their wild-type plasmids of GLS2 in HUH7 (left) and Hep3B (right) cells with knockdown of METTL14. **f** Relative luciferase activity of three mutant plasmids and their wild-type plasmids of GLS2 in HUH7 (left) and Hep3B (right) cells with overexpression of METTL14. **g** Western blot (left) and relative quantitative analysis (right) showing METTL14 and GLS2 expression in HUH7 cells infected with YTHDF1 overexpression or shRNA lentivirus vector. **h** Western blot (left) and relative quantitative analysis (right) showing METTL14 and GLS2 expression in Hep3B cells infected with YTHDF1 overexpression or shRNA lentivirus vector. **i** Relative luciferase activity of three mutant plasmids and their wild-type plasmids of GLS2 in HUH7 (left) and Hep3B (right) cells with knockdown of YTHDF1. **j** Relative luciferase activity of three mutant plasmids and their wild-type plasmids of GLS2 in HUH7 (left) and Hep3B (right) cells with overexpression of YTHDF1. **k** Western blot showing YTHDF1 and GLS2 expression in HUH7 (left) and Hep3B (right) cells transfected with YTHDF1 overexpression (oe-YTHDF1), YTHDF1 mutation (K395A & Y397A, Mut) and vector plasmid (Vector) respectively. **l** Relative luciferase activity of HUH7 (top) and Hep3B (bottom) cells transfected with YTHDF1 overexpression, YTHDF1 mutant (K395A and Y397A) and vector plasmids, respectively. **m** Western blot showing METTL14, YTHDF1 and GLS2 expression in HUH7 (left) cells and Hep3B cells (right) infected with YTHDF1 overexpression (oe-YTHDF1) and/or METTL14 shRNA (sh-METTL14) lentivirus vector as well as control vectors. Data are represented as mean ± SEM. NS not significant, *p < 0.05, **p < 0.01, ***p < 0.001, ****p < 0.0001

To clarify the relationship between METTL14 and GLS2 expression, we first generated overexpression or interference with METTL14 expression in HUH7 and Hep3B cells in vitro, and discovered that there was no significant change in *GLS2* mRNA levels, but GLS2 protein levels were consistently upregulated or downregulated (Fig. 3a and Supplementary Fig. 5a–d). To evaluate whether METTL14 could regulate the translation and degradation of GLS2 protein, we further treated cells with cycloheximide (CHX) to block protein translation or MG132 to inhibit proteasome activity. It was indicated that METTL14 interference or overexpression had no discernible effect on GLS2 protein levels in CHX-treated HUH7 cells (Supplementary Fig. 5e, f). MG132 could restore the level of GLS2 protein, however, the difference remained after interference or overexpression with METTL14 in HUH7 cells (Supplementary Fig. 5g, h). All these results indicated that METTL14 might regulate the protein translation rather than protein stability of GLS2.

As METTL14 is a pivotal m6A methyltransferase, we then detected the m6A modification level of *Gls2* mRNA in liver tissues of KO mice and HFD-fed mice, and found that the m6A modification level was significantly reduced in KO mice and HFD-fed mice as compared to their respective controls (Fig. 3b, c), suggesting that METTL14 might regulate GLS2 expression in an m6A-dependent manner. We then predicted the potential m6A binding sites of *GLS2* mRNA and identified the top three sites using the SRAMP database (Supplementary Table 4).^18^ To explore the potential roles of m6A modification on GLS2 expression, firstly, we constructed three wild-type fragment reporters containing the above m6A sites named GLS2 WT1/2/3, or mutants named MT1/2/3 (A/GGAC to A/GGCC, Fig. 3d) after the firefly luciferase reporter gene. The dual-luciferase assay showed the luciferase activities were decreased in HUH7 and Hep3B cells interfering METTL14, but were restored after transfected with GLS2 m6A mutants (Fig. 3e). Conversely, the luciferase activities were elevated in METTL14-overexpressed HUH7 and Hep3B cells, but were attenuated after transfected with GLS2 MT1/2/3 (Fig. 3f).

It was well-acknowledged that YTHDF1 could recognize m6A methylated mRNA and promote the translation of targeted mRNA.^19^ In order to explore the potential involvement of YTHDF1 in the regulation of m6A modified GLS2 expression mediated by METTL14, we further detected the expression of GLS2 in HUH7 and Hep3B cells with YTHDF1 interference or overexpression. It was shown that GLS2 was consistently upregulated or downregulated in HUH7 and Hep3B cells with YTHDF1 overexpression or interference (Fig. 3g, h). The dual-luciferase assay showed the luciferase activities were decreased in YTHDF1-interfered HUH7 and Hep3B cells, but were restored after transfected with GLS2 m6A mutants (Fig. 3i). Conversely, the luciferase activities were increased in HUH7 and Hep3B cells overexpressing YTHDF1, but were attenuated after transfected with GLS2 m6A mutants (Fig. 3j). YTHDF1 is known to bind m6A sites via its m6A-binding pockets in the YTH domain; mutations in K395 and Y397 could eliminate the mRNA-binding capacity of YTHDF1.^20,21^ We then constructed YTHDF1-overexpressed plasmid (oe-YTHDF1-wt) as well as its mutant by introducing two-point mutations K395A and Y397A in YTH domain of YTHDF1 (oe-YTHDF1-mut) and transfected them into HUH7 and Hep3B cells, respectively (Supplementary Fig. 5i). Subsequently, RIP-PCR analysis by using an anti-YTHDF1 antibody revealed that *GLS2* mRNA was immunoprecipitated effectively in HUH7 and Hep3B cells transfected with YTHDF1-wt, but the interaction between YTHDF1 mutants and *GLS2* mRNA was drastically decreased (Supplementary Fig. 5j). GLS2 protein levels changed in the same way (Fig. 3k and Supplementary Fig. 5k). Moreover, the luciferase activities were increased in HUH7 and Hep3B cells co-transfected with GLS2 WT 1/2/3 plasmids and oe-YTHDF1-wt construct, but were attenuated when co-transfected with oe-YTHDF1-mut construct (Fig. 3l), suggesting the m6A-binding pocket were crucial for YTHDF1 to bind *GLS2* mRNA. Furthermore, GLS2 expression was rescued when METTL14-interfered HUH7 and Hep3B cells were treated with YTHDF1 overexpression (Fig. 3m and Supplementary Fig. 5l).

Taken together, these results suggested that METTL14 regulated the protein translation of GLS2 mediated by the m6A reader YTHDF1 in an m6A-dependent manner.

### Hepatocyte *Mettl14* deficiency resulted in an increase in *Cx3cr1*^+^*Ccr2*^+^ Mo-macs in liver tissue

**Fig. 4:**
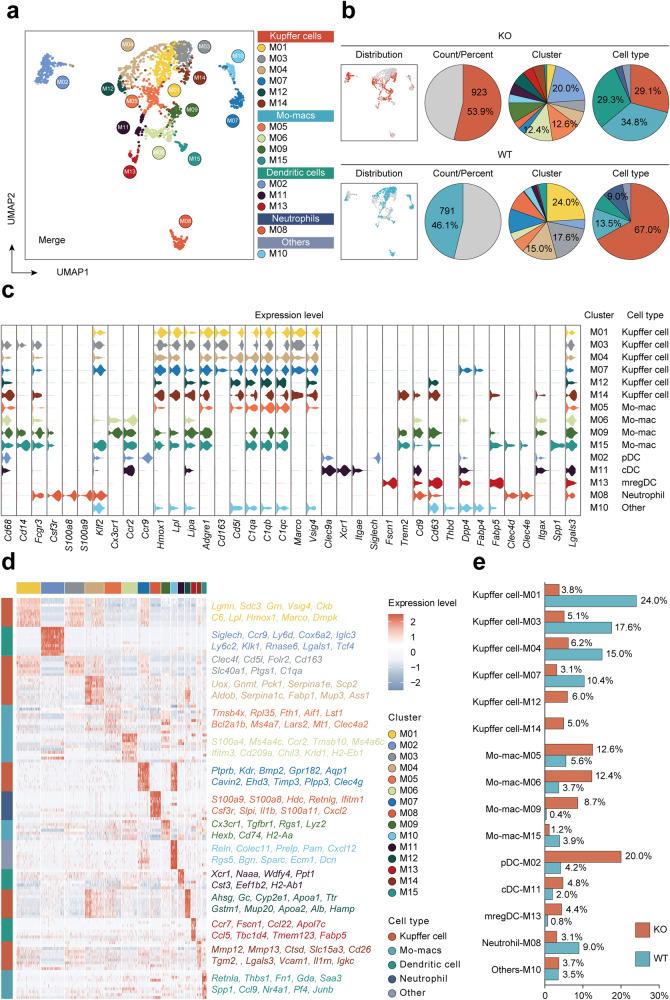
Increased abundance of *Cx3cr1*^+^*Ccr2*^+^ macrophages in the liver of the KO group. **a** UMAP plot displaying the distributions of 6 Kupffer cell clusters, 4 Mo-mac clusters, 3 dendritic cell clusters, 1 neutrophil cluster and 1 other cluster. **b** The percentage of cell counts, cell clusters and cell types in the KO and WT groups. **c** Violin plots showing marker genes and markers of lipid associated macrophages (LAMs) or scar associated macrophages (SAMs) across each cluster. **d** DEGs for Kupffer cells, monocyte-derived macrophages, dendritic cells and neutrophil clusters. **e** Bar plot showing different proportions of each cluster in the KO and WT groups

Previous studies have shown that GLS2 functions as an antioxidant by catalyzing the hydrolysis of glutamine to produce glutamic acid and increasing reduced glutathione (GSH) levels.^14,15^ The low expression of GLS2 in hepatocytes leads to the accumulation of reactive oxygen species (ROS), which may lead to the release of damage-associated molecular pattern molecules (DAMPs).^16^ DAMPs are cell-derived molecules that respond to trauma, local ischemia, and tissue damage in the absence or presence of pathogenic infections, initiating and maintaining immunity.^22^ Macrophages are believed to play an important role in the initiation and spread of liver inflammation, as well as in the regulation of liver fibrosis.^23^ Considering that the oxidative stress microenvironment of liver tissue is the basic feature of MAFLD-related liver tissue and the key factor in fostering the progression of inflammation, non-parenchymal cells of one KO and one WT mouse liver were used for single-cell RNA sequencing (scRNA-seq).^24,25^ A total of 14374 individual non-parenchymal cells were included in the analysis at 18548 gene feature levels after quality control. Given the key role of macrophages in the progression of MAFLD, we focused on the composition changes of myeloid cells between KO and WT mice.^23,26^ To determine the expression features of various cell types, all the myeloid cells were classified into fifteen clusters and five specific cell types (Fig. 4a, b). We then displayed the marker genes and differentially expressed genes (DEGs) among each cell cluster from different cell types (Fig. 4c, d). The results of cell counting showed that KCs decreased significantly in KO mice (M01, M03, M04 and M07), while Mo-macs instead increased significantly (M05, M06 and M09) (Fig. 4e). M05 cluster comprises 12.6% of myeloid cells in KO mice and 5.6% in WT mice. We then performed differentially expressed gene analysis and presented the top 20 genes among M05 cluster (Supplementary Fig. 6a). GO and KEGG analysis further revealed that this cluster of Mo-macs was associated with ribosome activity and translation process (Supplementary Fig. 6b). Interestingly, we discovered that M06 and M09 clusters are characterized by high expression of *Cx3cr1* and *Ccr2* (Fig. 4c). To confirm this subset of cells, we labeled CX3CR1 and CCR2 double-positive cells in the liver tissues of KO and WT mice and discovered that CX3CR1^+^CCR2^+^ cells were markedly increased in the liver tissues of KO mice (Supplementary Fig. 7a). More importantly, CX3CR1^+^CCR2^+^ cells were consistently accumulated in the liver tissues of HFD mice when compared to those of control diet mice (Supplementary Fig. 7b), suggesting that these cells might play an important role in the progression of MAFLD.^27–29^ We also noticed that M09 and M15 Mo-macs also remarkably expressed specific markers of lipid-associated macrophages (LAMs) or scar-associated macrophages (SAMs) including *Trem2*, *Gpnmb*, *Cd9*, *Cd63*, *Cx3cr1*, *Ccr2*, and *Fabp5*, which have been documented to play pivotal roles in the progression of MAFLD.^2,28,30–33^ It was possible that M09 and M15 were a typical cluster of either SAMs or LAMs.

**Fig. 5:**
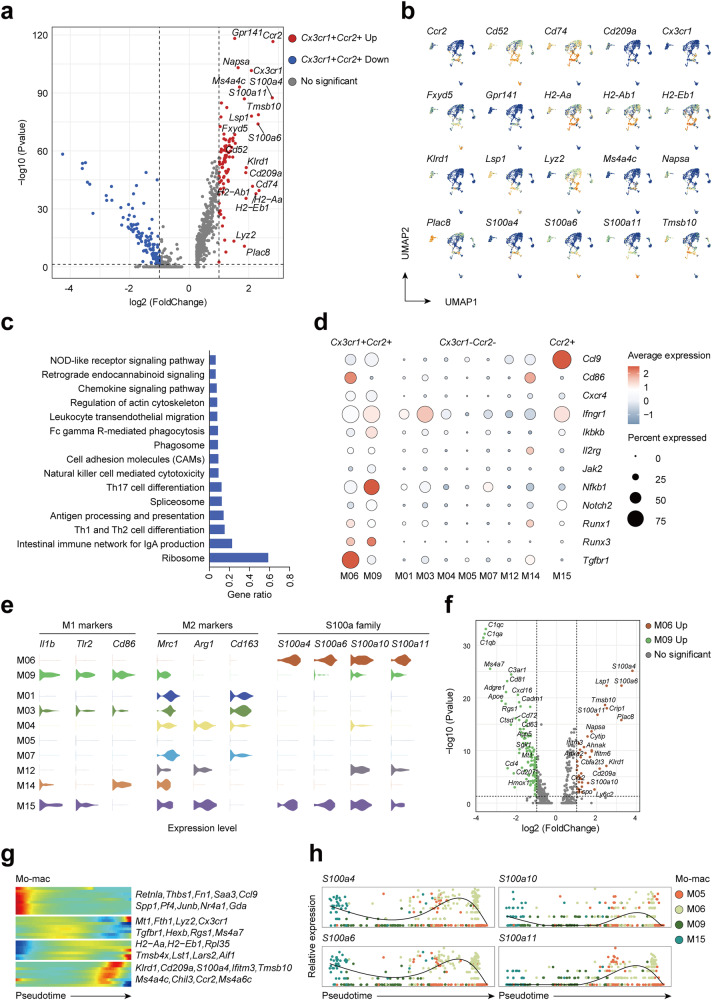
*Cx3cr1*^+^*Ccr2*^+^ Mo-macs in the late stage of the developmental trajectory highly expressed S100 family protein genes. **a** Volcano plot comparing DEGs for *Cx3cr1*^+^*Ccr2*^+^ Mo-macs. **b** Feature plots showing the top 20 DE genes. **c** KEGG pathway enrichment analysis of upregulated DE genes in *Cx3cr1*^+^*Ccr2*^+^ Mo-macs. **d** Dot plot presenting the expression strength of key KEGG pathway genes among *Cx3cr1*^+^*Ccr2*^+^, *Cx3cr1*^-^*Ccr2*^-^and *Ccr2*^+^ Mo-macs and Kupffer cells. **e** Violin plots displaying the expression levels of polarization state marker genes and S100a family protein genes. **f** Volcano plot comparing DEGs between M06 and M09. **g** Heatmap displaying the expression of selected marker genes in Mo-macs arranged along the pseudotime trajectory. **h** Scatter plots and fitting curves presenting the expression trend of *S100a4*, *S100a6*, *S100a10* and *S100a11*

To explore the roles of increased *Cx3cr1*^+^*Ccr2*^+^ Mo-macs in *Mettl14* deficiency and MAFLD progression, we further analyzed the gene expression patterns of *Cx3cr1*^+^*Ccr2*^+^ Mo-macs. We displayed the top 20 differentially upregulated genes of *Cx3cr1*^+^*Ccr2*^+^ Mo-macs (Fig. 5a, b), which imply that these macrophages have the capacity to promote inflammatory progression and subsequent fibrosis. For example, pharmacological inhibition of CCR2^+^ monocyte recruitment efficiently ameliorates hepatic inflammation and fibrosis in patients with metabolic steatohepatitis.^34^ S100A4 derived from macrophages in the liver promotes liver fibrosis through hepatic stellate cell activation.^35^ Pathway analysis further revealed that *Cx3cr1*^+^*Ccr2*^+^ Mo-macs were enriched for inflammatory pathways and inflammation-activated key genes (Fig. 5c, d). When further analyzing the characteristic expression genes of M06 and M09 clusters, it was discovered that M09 highly expressed M1 macrophage-related markers (*Il1b*, *Tlr2* and *Cd86*), whereas M06 expressed neither M1 nor M2 macrophage markers (*Mrc1*, *Arg1* and *Cd163*). Strikingly, M06 expressed high levels of *S100a4, S100a6, S100a10* and *S100a11* (Fig. 5e, f). These findings imply that despite the fact that both the M06 and M09 subsets are *Cx3cr1*^+^*Ccr2*^+^ Mo-macs, their contributions to the progression of inflammation may be distinct.

Furthermore, we visualized the transcriptional profile of Mo-macs and mapped them along pseudotime trajectories (Supplementary Fig. 8a). Our data implied that *Cx3cr1*^+^*Ccr2*^+^ macrophages were in the late stage of the developmental trajectory (Supplementary Fig. 8b). Moreover, the cells from KO mice were located in the pseudotime trajectories later than those from WT mice (Supplementary Fig. 8b). To gain insights into the differentiation process of *Cx3cr1*^+^*Ccr2*^+^ Mo-macs, we generated a branched heatmap to decipher the gene expression patterns of M06 and M09 on the dynamics of DEGs (Fig. 5g). These genes were then categorized into six groups according to their characterized patterns, and multiple S100a family members (*S100a4, S100a6, S100a10* and *S100a11*) were evidently present at the end of the pseudotime trajectory among the M06 cluster (Fig. 5g, h). S100A proteins are calcium-binding proteins that are commonly dysregulated in various diseases, including MAFLD.^36,37^ HFD increased liver S100A11 expression, which may interact with HDAC6, disrupt its binding to FoxO1, release or enhance FoxO1 acetylation, and thus accelerate lipid accumulation and hepatic steatosis.^37^ A recent study showed that macrophage-derived S100A4 can promote liver fibrosis by activating HSCs in a CCl_4_-induced liver fibrosis model.^35^ Therefore, we had reason to believe that the high expression of S100A proteins in *Cx3cr1*^+^*Ccr2*^+^ Mo-macs might play a crucial role in activating HSCs in the progression of MAFLD.

### S100A4 expression was activated by CX3CR1/MyD88/NF-κB pathway in *Cx3cr1*^+^*Ccr2*^+^ Mo-macs

It was discovered that the levels of ROS increased while antioxidant capacity decreased in hepatocytes of KO mice, indicating increased oxidative stress levels within the hepatic microenvironment (Fig. 2j, k). Moreover, KO mice displayed an elevation in the presence of damage-associated molecular patterns (DAMPs), such as 8-OHdG (Fig. 2l), as well as accumulation of *Cx3cr1*^+^*Ccr2*^+^ Mo-macs that expressed S100A4 (Figs. 4, 5). DAMPs are known to be released from impaired hepatocytes, recognized by pattern-recognition receptors (PRRs) and then start a highly optimized order of immune cell recruitment such as monocytes and neutrophils to initiate inflammatory response.^38–41^ The Toll-like receptors (TLRs), as prominent PRRs for DAMPs, have the ability to dimerize and to undergo conformation changes, resulting in the recruitment of TLR domain-containing adaptor proteins such as myeloid differentiation factor 88 (MyD88).^42^

**Fig. 6:**
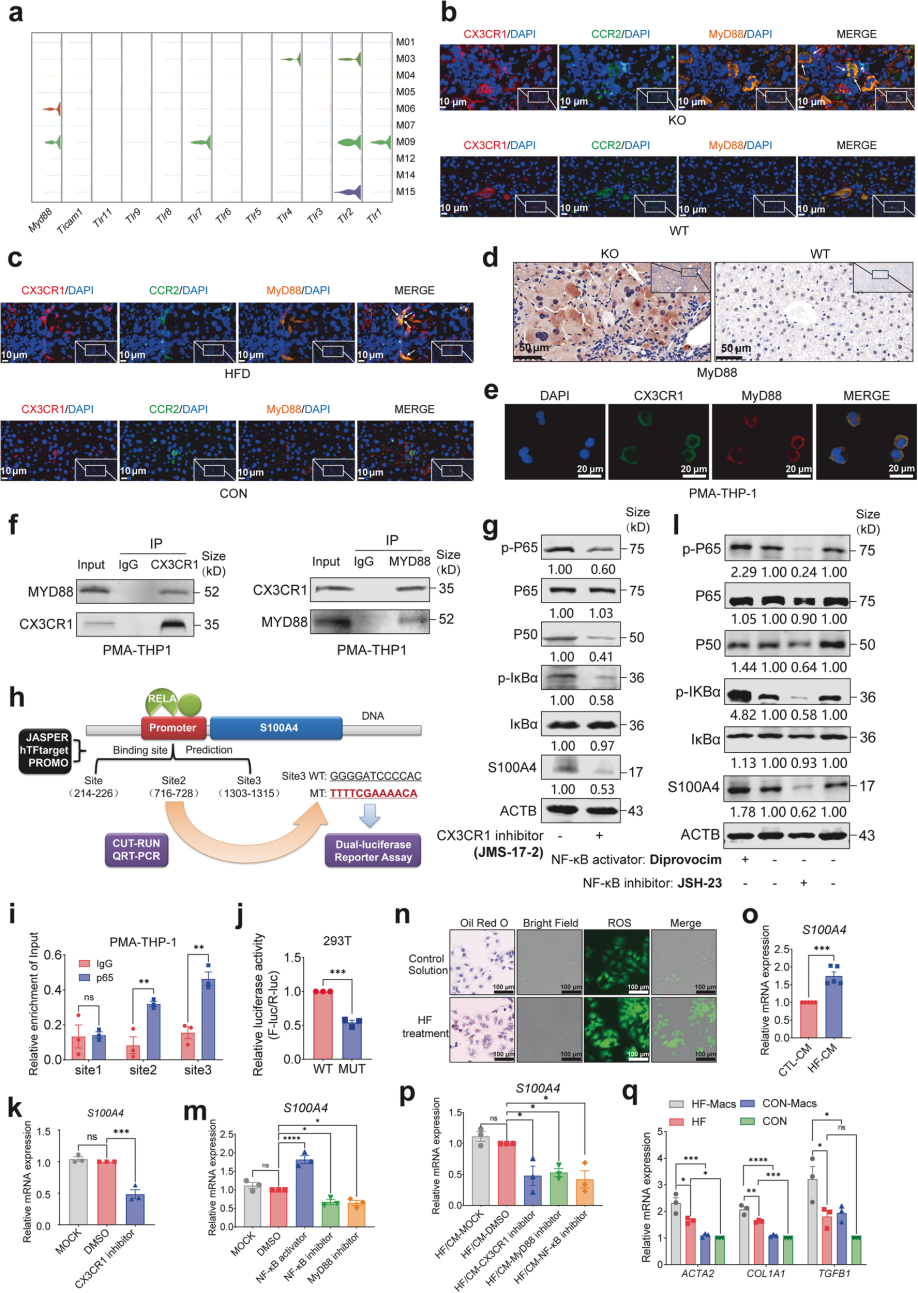
CX3CR1/MYD88/NF-κB regulated the expression of S100A4 in MAFLD progression. **a** Violin plots showing the expression of Toll-like receptors (TLRs) and adaptor MYD88 across each myeloid cell cluster. **b**, **c** Immunofluorescence analysis showing the distributions of CX3CR1^+^CCR2^+^MYD88^+^ cells in the KO and WT mice, HFD-fed and control diet-fed mice (*n* = 3, scar bar = 10 μm). **d** Immunohistochemical staining analysis of MYD88 in WT and KO mice (*n* = 3, scar bar = 50 μm). **e** Immunofluorescence analysis showing the colocalization of CX3CR1 and MYD88 in PMA-treated THP-1 cells by confocal fluorescence microscopy (scar bar = 20 μm). **f** Co-Immunoprecipitation analysis showing interaction between CX3CR1 and MYD88 in PMA-treated THP-1 cells. **g** Western blot showing the expression of NF-κB family proteins and S100A4 after treated with CX3CR1 inhibitor JMS-17-2. **h** Schematic diagram showing the procedures of RELA (P65) binding to the promoter of S100A4 and subsequent assays. **i** CUT-RUN analysis showing the binding capacity of RELA (p65) on three binding sites of S100A4 promoter regions in PMA-treated THP-1 cells. **j** Relative luciferase activity of mutant plasmid and wild-type plasmid of RELA binding sequences (site 3 in h) in 293T cells. **k** QRT-PCR analysis indicating mRNA expression of *S100A4* after treated with CX3CR1 inhibitor JMS-17-2 in PMA-treated THP-1 cells. **l** Western blot showing the expression of NF-κB family proteins and S100A4 after treated with Diprovocim (NF-κB activator) or JSH-23 (NF-κB inhibitor). **m** QRT-PCR analysis showing *S100A4* mRNA expression after treated with NF-κB activator/inhibitor and MyD88 inhibitor (ST2825) in PMA-treated THP-1 cells. **n** Oil red O staining and ROS analysis of HUH7 cells with HF treatment (12 μmol/l oleic acid and 6 μmol/l palmitic acid) and control solution for 48 h (scar bar = 100 μm). **o** QRT-PCR analysis showing the expression of S100A4 of PMA-treated THP-1 cells after cultured by HF conditional medium for 24 h. **p** Rescue assay showing the expression of S100A4 in PMA-treated THP-1 cells after cultured by HF conditional medium with/without CX3CR1 inhibitor, MyD88 inhibitor or NF-κB inhibitor. **q** QRT-PCR analysis showing the expression of HSC activation markers such as *ACTA2*, *COL1A1*, and *TGFB1* of LX2 cells with/without high-fat supernatant stimulation as well as cocultivation with monocyte-derived macrophages (Mo-macs). Data are represented as mean ± SEM. NS not significant, *p < 0.05, **p < 0.01, ***p < 0.001, ****p < 0.0001

To investigate why *Cx3cr1*^+^*Ccr2*^+^ Mo-macs accumulate in KO mice, as well as the cause of their S100A4 expression, we analyzed the expression of TLRs and related adaptor proteins in the subsets of myeloid cells (Fig. 6a). Strikingly, *Myd88* was expressed in both M06 and M09 clusters of *Cx3cr1*^+^*Ccr2*^+^ Mo-macs, while *Tlr1*, *Tlr2* and *Tlr7* were only expressed in M09 clusters that expressed M1-like macrophage markers (Fig. 6a). We then labeled CX3CR1, CCR2 and MyD88 triple-positive cells in the liver tissues of KO and WT mice by tissue immunofluorescence analysis. The results showed that CX3CR1^+^CCR2^+^MyD88^+^ cells were remarkably increased in the liver tissues of KO mice (Fig. 6b). More importantly, CX3CR1^+^CCR2^+^MyD88^+^ cells were also increased in the liver tissues of HFD mice (Fig. 6c). The expression of MyD88 was also increased in the KO mice (Fig. 6d). MyD88 has been found to be elevated in the liver tissues of MASH patients and MAFLD murine models.^43,44^ MyD88 deficiency substantially decreases lipid accumulation and liver fibrosis.^44,45^ TLRs, except for TLR3, have been extensively reported to use MyD88 to activate transcription factors including nuclear factor-κB (NF-κB), activator protein 1 (AP-1), and interferon regulatory factors that generate inflammatory cytokines, which was consistent with the expression pattern of M09 cluster (Fig. 6a).^46,47^ However, considering the expression features of M06 cluster, which lacked expression of TLRs and M1/M2-like macrophage markers, we hypothesized that DAMPs could activate downstream MyD88/NF-κB inflammatory signaling pathways via CX3CR1 or CCR2 in Mo-macs. To test this hypothesis, we first developed an in vitro macrophage model by using phorbol-12-myristate-13-acetate (PMA)-treated monocyte THP-1 cells.^48^ Then we stained PMA-stimulated THP-1 cells with anti-CX3CR1 and anti-MyD88 antibodies, and observed the colocalization of CX3CR1 and MyD88 protein by confocal microscopy (Fig. 6e). Co-immunoprecipitation (Co-IP) assays further revealed that endogenous CX3CR1 colocalized with MyD88 and that CX3CR1 was pulled down by endogenous MyD88 (Fig. 6f). To evaluate whether CX3CR1 could affect the downstream signaling pathway of MyD88, such as classical NF-κB signaling pathway, we treated PMA-treated THP-1 cells with CX3CR1 inhibitor JMS-17-2 and detected the signaling pathway activation level of NF-κB signaling pathway. It was discovered that though total P65 and IκBα did not change significantly, phosphorylated P65, phosphorylated IκBα as well as P50 were decreased dramatically after CX3CR1 inhibitor treatment (Fig. 6g).

It has been discovered that S100A4 is expressed by a subpopulation of macrophages in the fibrotic liver and extracellular S100A4 serves as a fibrotic factor through activating HSCs.^35^ In our studies, we discovered that a subset of CX3CR1^+^CCR2^+^MYD88^+^ Mo-macs expressed S100A4; however, the reason for the increased expression of S100A4 in macrophages has not been established. Since extracellular S100A4 can promote the NF-κB activation and consequently induce an inflammatory response^49,50^ and the fact that CX3CR1 could regulate MyD88/NF-κB signaling pathway (Fig. 6g), we hypothesized that NF-κB transcription regulators could conversely activate S100A4 transcriptional expression in macrophages and exhibit a mutually reinforcing regulatory interaction with S100A4 that collectively enhances the course of inflammation. Therefore, we subsequently predicted the potential binding sites of the NF-κB family transcription factors in the S100A4 promoter region using three online databases including JASPER, hTFtarget and PROMO (Fig. 6h).^51–53^ Cleavage under target and release using nuclease (CUT-RUN) and QRT-PCR analysis indicated that the third predicted site exhibited the highest level of enrichment using the anti-P65 antibody in PMA-treated THP-1 cells (Fig. 6i). We next constructed the plasmid vector including the DNA fragments of the third site as well as corresponding mutant plasmid. Dual-luciferase assay revealed the decreased luciferase activities when transfected with mutant plasmid in 293 T cells (Fig. 6j), indicating that NF-κB could affect the transcription activity of S100A4 promoter. Furthermore, it was discovered that the mRNA and protein levels of S100A4 were reduced in PMA-induced THP-1 cells treated with CX3CR1 inhibitor (Fig. 6k, g). Subsequently, we treated with PMA-stimulated THP-1 cells with an activator (Diprovocim) or an inhibitor (JSH-23) of NF-κB signaling pathway, and the protein levels of phosphorylated P65, phosphorylated IκBα as well as P50 were elevated or diminished as expected, accompanied by a drop in the protein level of S100A4 (Fig. 6l). The mRNA level of *S100A4* was likewise affected by NF-κB activator or inhibitor treatment, as well as decreased by MyD88 inhibitor ST2825 treatment (Fig. 6m). To elucidate the role of CX3CR1/MyD88/NF-κB signaling pathway in regulating the expression of S100A4 during MAFLD progression, we cultured HUH7 cells with oleic acid and palmitic acid to generate high-fat cell models in vitro and validated the lipid accumulation and oxidative stress state in cell models using Oil Red O staining and ROS detection (Fig. 6n). The culture supernatant from high-fat cells was collected and used as a conditional medium to culture PMA-treated THP-1 cells. It was discovered that conditional medium derived from high-fat cells could activate S100A4 expression (Fig. 6o), and further treatments with either a CX3CR1 inhibitor, a MyD88 inhibitor or a NF-κB inhibitor could conversely reduce the levels of S100A4 (Fig. 6p).

To further verify the indispensable roles of these monocyte-derived macrophages, we cultured LX2 cells using centrifugated supernatants from high fat (HF) treated cells that were either co-cultured or not with THP-1 induced monocyte-derived macrophages in vitro and detected the expression of activated LX2 markers, including *COL1A1*, *ACTA2*, and *TGF-β1*. The results showed that the activation levels of LX2 were higher in the presence of Mo-macs (Fig. 6q). Collectively, these findings suggest that CX3CR1/MyD88/NF-κB pathway could regulate the expression of S100A4.

### Blocking MyD88 signal alleviates liver inflammation, injury and fibrosis in *Mettl14* deficient mice

Using scRNA-seq data of liver non-parenchymal cells, our analysis strategy re-clustered a subset of 331 HSCs into three distinct subpopulations (HSC01, HSC02 and HSC03) (Supplementary Fig. 9a). The analysis of the proportion of each HSC subgroup showed that the proportion of HSC03 in the KO mouse sample increased significantly (Supplementary Fig. 9a), which specifically expressed key activation marker genes such as *Col1a1*, *Col1a2*, *Col3a1* and *Col6a3* (Supplementary Fig. 9b, c).

To determine the inflammatory-related factors regulated by hepatocyte *Mettl14* deficiency, we performed cytokine protein array analysis to identify changes in inflammatory-related factor expression levels in mouse livers following hepatocyte *Mettl14* deficiency. Of the 200 inflammatory-related factors examined, 36 were significantly upregulated in KO mice (Supplementary Fig. 9d). To determine which categories of cells express these upregulated inflammatory-related factors in KO mice, we displayed the levels of these genes in nonparenchymal cells using scRNA-seq data (Supplementary Fig. 9e). The results demonstrated that most of these upregulated inflammatory-related factors originated from myeloid cells and HSCs (Supplementary Fig. 9e). Also, cytokines associated with stellate cell activation and fibrosis promotion, such as *Hgf*,^54^ *Mmp2*^55^ and *Fgf2*,^56^ were highly expressed in the HSC03 cluster (Supplementary Fig. 9e), indicating that HSC03 may be an activated HSC in *Mettl14* deficient mice. An enzyme-linked immunosorbent assay (ELISA) of liver tissues also confirmed the elevated levels of HGF, MMP2, FGF2 and S100A4 in the KO mice compared to those of WT mice (Supplementary Fig. 9f–i). Furthermore, we observed increased expression of intrahepatic α-SMA (a marker of HSC activation) in KO mice (Supplementary Fig. 9j, k). Meanwhile, Sirius red staining demonstrated that collagen deposition in KO mice was significantly higher than in WT mice (Supplementary Fig. 9l, m). Collectively, these results suggested that the proportion of activated HSCs and degree of fibrosis in the liver tissue of KO mice may be much more severe than those of WT mice.

To further clarify the roles of CX3CR1/MyD88/NF-κB pathway in liver inflammation activation and fibrosis processes, we subsequently evaluated the rescued function of MyD88 inhibitor ST2825 in *Mettl14* deficient mice. KO mice were intraperitoneally injected with ST2825 or corn oil once a week for two months (Supplementary Fig. 10a). KO mice with oil treatment displayed more irregular or inflammatory liver nodules than KO mice with ST2825 treatment (Supplementary Fig. 10b). And KO mice treated with ST2825 gained weight more rapidly and had a lower ratio of liver to body weight than KO mice with oil (Supplementary Fig. 10c, d). Serum levels of ALT, AST and total bilirubin (TBIL) were alleviated in KO mice with ST2825 treatment (Supplementary Fig. 10e), as were serum S1000A4 levels (Supplementary Fig. 10f). Furthermore, the fibrotic markers of *Col1a1*, *Acta2* and *Mmp2* were decreased, and collagen deposition and the activation of HSCs were reduced in KO mice treated with ST2825 (Supplementary Fig. 10g, h). Collectively, these findings further demonstrate that inhibiting MyD88 in *Mettl14-*deficient mice could rescue the liver injury, inflammation and fibrosis, highlighting the crucial roles of *Cx3cr1*^*+*^*Ccr2*^*+*^ Mo-macs cells with high expression of MyD88 in the process of liver inflammation and fibrosis.

### Restoration of METTL14 or GLS2 ameliorates the progress of MAFLD

**Fig. 7:**
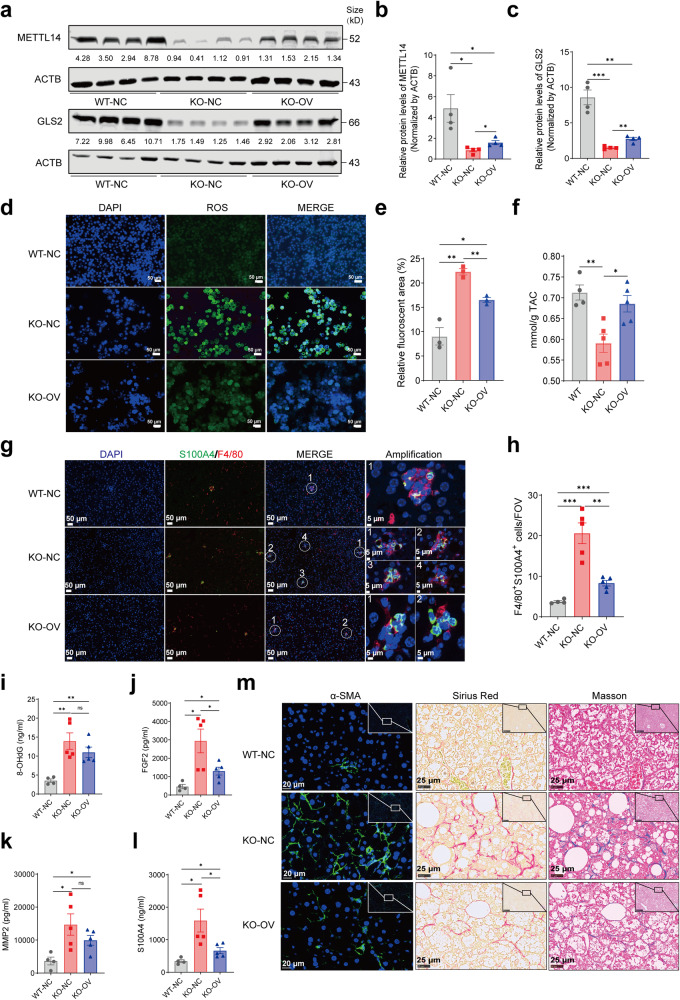
Restoration of METTL14 function in mice ameliorates liver inflammation, injury, and fibrosis. **a** Western blot showing METTL14 and GLS2 expression in mouse livers in the WT-NC, KO-NC, and KO-OV groups (*n* = 4). **b**, **c** Relative quantitative analysis of METTL14 (**b**) and GLS2 (**c**) expression in mouse livers in the WT-NC, KO-NC, and KO-OV groups. **d** DCFH-DA was used to display the levels of intracellular ROS in primary cultured hepatocytes isolated from WT-NC, KO-NC, and KO-OV mouse livers (*n* = 3, scar bar = 50 μm). **e** Relative quantitative analysis of ROS levels in primary cultured hepatocytes isolated from WT-NC, KO-NC, and KO-OV mouse livers. **f** TAC levels showing the total antioxidant capacity of mouse livers using ABTS methods in the WT-NC (*n* = 4), KO-NC (*n* = 5), and KO-OV (*n* = 5) groups. **g** Representative immunofluorescence images displaying the distributions of S100A4-positive, F4/80-positive, and S100A4 F4/80 double-positive macrophages among liver sections in the WT-NC, KO-NC, and KO-OV groups (scar bar = 50 μm or 5 μm). **h** Relative quantitative analysis of S100A4 F4/80 double-positive macrophages in the WT-NC (*n* = 4), KO-NC (*n* = 5), and KO-OV (*n* = 5) groups. FOV, field of view. **i**–**l** ELISA array detecting the levels of 8-OHdG (**i**), FGF2 (**j**), MMP2 (**k**), and S100A4 (**l**) in mouse liver tissues from the WT-NC (*n* = 4), KO-NC (*n* = 5), and KO-OV (*n* = 5) groups. **m** Immunofluorescence staining detecting α-SMA expression (left), Sirius Red staining (middle) and Masson staining (right) displaying the activation of HSCs and the severity of fibrosis of liver sections in the WT-NC (*n* = 4), KO-NC (*n* = 5), and KO-OV (*n* = 5) groups (scar bar = 20 μm or 25 μm). Data are represented as mean ± SEM. NS not significant, *p < 0.05, **p < 0.01, *** *p* < 0.001

In order to further clarify the role of low expression of METTL14 in liver inflammation and fibrosis, we restored METTL14 in HFD-treated KO and WT mice via liver-specific AAVs overexpressing METTL14 (Fig. 1i). We confirmed the restoration of METTL14 as well as the upregulated GLS2 at the protein level (Fig. 7a–c). Previous findings indicated that the expression of GLS2 was comparably reduced in KO or HFD mice. Consequently, we investigated whether the expression of GLS2 was further diminished in HFD-fed KO mice. It was found that the levels of GLS2 in HFD-fed KO mice did not differ significantly from those of KO mice of comparable age fed the control diet (Supplementary Fig. 11a). Subsequent investigations confirmed a decrease in the levels of ROS in the hepatocytes of KO-OV mice (Fig. 7d, e), and the antioxidant capacity was elevated dramatically when compared with KO-NC mice (Fig. 7f). These results strongly supported that METTL14 could regulate the expression of GLS2 and subsequently modulate the oxidative stress microenvironment in liver tissues, which was further validated by the decreasing tendency of 8-OHdG levels in KO-OV mice (Fig. 7i). Additionally, whereas F4/80^+^ positive cells accumulated in the liver tissues of KO-OV mice, S100A4^+^ or F4/80^+^S100A4^+^ cells conversely diminished in the KO-OV mice (Fig. 7g, h and Supplementary Fig. 11b, c), which confirmed causal effects of cell composition alterations of macrophages such as Mo-macs from the hepatocyte-specific depletion or hepatic restoration of METTL14. Subsequently, we discovered a conspicuous drop in FGF2 and S100A4 levels and a slight decrease in the levels of MMP2 in the KO-OV mice (Fig. 7j–l). We also observed a decrease in the activation of HSCs by detecting the protein levels of α-SMA and a significant decline in the fibrosis degree in the KO-OV mice (Fig. 7m and Supplementary Fig. 11d–f), revealing that restoration of METTL14 could alleviate liver injuries, inflammation and fibrosis in the livers of KO-OV mice during the progression of MAFLD.

**Fig. 8:**
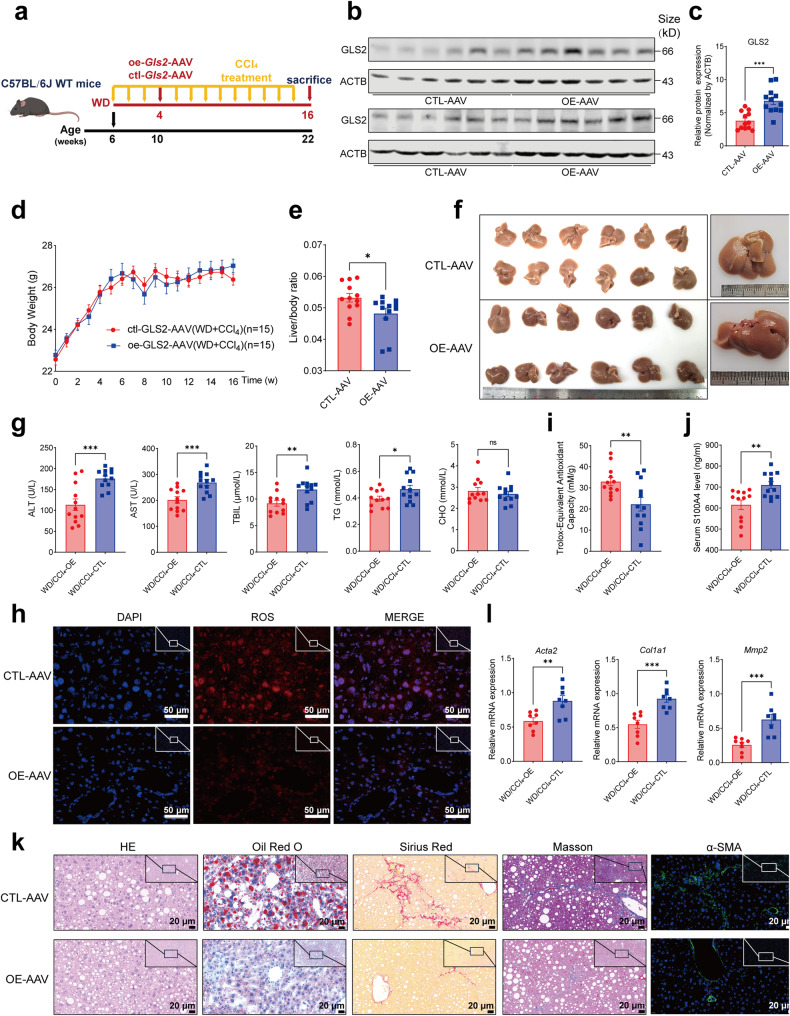
Restoration of GLS2 ameliorates liver inflammation, injury, and fibrosis in Western diet/CCl_4_-treated mice. **a** Schematic diagram of the dietary feeding scheme. Six-week-old C57/BL6 wild-type mice were fed a Western diet (WD) for 16 weeks and also treated with CCl_4_ intraperitoneal injection. Mice fed a WD for 4 weeks were randomly injected with AAV-8 overexpressing *Gls2* (OE-AAV) or control vector (CTL-AAV) through the caudal vein (*n* = 15). **b**, **c** Western blot (**b**) and quantitative analysis (**c**) showing the expression of GLS2 in the OE-AAV and CTL-AAV mice (*n* = 12). **d** Body weight changes of OE-AAV and CTL-AAV mice (*n* = 15). **e** Comparison of the liver-to-body weight ratio of OE-AAV and CTL-AAV mice (*n* = 12). **f** Representative image of resected livers from OE-AAV and CTL-AAV mice (*n* = 12). **g** Serum ALT, AST, TBIL, TG and CHO in OE-AAV and CTL-AAV mice (*n* = 12). **h** Tissue ROS analysis of liver sections from OE-AAV and CTL-AAV mice (*n* = 4, scar bar = 50 μm). **i** Total antioxidant capacity analysis of OE-AAV and CTL-AAV mice (*n* = 12). **j** ELISA analysis showing serum S100A4 levels of OE-AAV and CTL-AAV mice (*n* = 12). **k** HE, Oil Red O, Sirius Red, Masson staining and α-SMA immunofluorescence analysis of OE-AAV and CTL-AAV mice (*n* = 12, scar bar = 20 μm). **l** QRT-PCR analysis showing the expression of fibrosis markers such as *Acta2*, *Col1a1* and *Mmp2* of OE-AAV and CTL-AAV mice (*n* = 8). Data are represented as mean ± SEM. NS not significant, *p < 0.05, **p < 0.01, ***p < 0.001. The mouse image was created with BioRender.com

Considering that the reduced expression of GLS2 was a key link in the promotion of liver inflammation and fibrosis by hepatocyte METTL14 deficiency (Supplementary Fig. 4), during the progression of MAFLD, we conducted a MASH mouse model using a WD diet plus CCl_4_ treatment for 16 weeks (Fig. 8a). Serotype 8 AAVs overexpressing *Gls2* (OE-AAV) or control vectors (CTL-AAV) were injected into WD/CCl_4_-treated mice via caudal veins four weeks after treatment began (Fig. 8a) and GLS2 overexpression was confirmed in the OE-AAV mice (Fig. 8b, c). Although there was no significant difference in body weight change between the OE-AAV and CTL-AAV groups (Fig. 8d), the ratio of liver weight to body weight was significantly lower (Fig. 8e), and morphological changes of resected livers appeared to have lower fat infiltration in the OE-AAV mice (Fig. 8f). Serum ALT, AST, TBIL, and TG levels were reduced during MAFLD progression (Fig. 8g). Furthermore, mice with GLS2 overexpression had lower ROS levels and higher total antioxidant capacity (Fig. 8h, i). The level of serum S100A4 was reduced, which was in line with the changes observed in overexpressing Gls2 in KO mice (Fig. 8j and Supplementary Fig. 4n) and accompanied by a decrease in α-SMA, a marker of HSC activation (Fig. 8k). Furthermore, lipid droplet accumulation and collagen deposition were reduced in the OE-AAV mice, which was confirmed by downregulated fibrotic markers such as *Col1a1*, *Acta2*, and *Mmp2* (Fig. 8k, l). Collectively, these results demonstrated that restoration of GLS2 ameliorates liver inflammation, injury, and fibrosis in mice with MAFLD progression.

Our work reveals the METTL14 regulates the expression of GLS2 in an m6A-dependent manner and facilitates an oxidative stress microenvironment that recruites *Cx3cr1*^+^*Ccr2*^+^ Mo-macs. These cells further activated HSCs via CX3CR1/MyD88/NF-κB pathway and consequently promoted liver fibrosis and MAFLD progression. In order to ascertain whether this regulatory mechanism is particular to MAFLD or general to liver pathogenesis such as viral hepatitis-induced liver fibrosis, we subsequently detected these regulating molecules (METTL14, GLS2, MyD88, S100A4, and α-SMA) and fibrosis degrees in the para-tumor tissues from the patients with HBV infection (Supplementary Fig. 12a–f). The results showed that the expression level of METTL14 was lower in liver tissue with a high degree of fibrosis, coinciding with elevated levels of MyD88, S100A4 and α-SMA, while there was no difference in the expression level of GLS2 (Supplementary Fig. 12c). These results indicated that this mechanism was specific to MAFLD, not general to liver pathogenesis.

## Discussion

The current study showed that *Cx3cr1*^+^ *Ccr2*^+^ Mo-macs expressed the adaptor *Myd88* and activated the transcription of S100A4 via CX3CR1/MyD88/NF-κB signaling pathway, resulting in the activation of HSCs and thereby promoting the progression of inflammation and liver fibrosis. More importantly, restoration of METTL14, GLS2 or MyD88 inhibition could ameliorate liver injuries, inflammation and fibrosis, which might bright light to the treatment of MAFLD.

During the evolution of steatosis-MASH-hepatic fibrosis, hepatocytes experience a series of important molecular events, such as somatic mutation selection,^57^ accumulation of epigenetic modifications, and changes in the dynamic signal regulation network.^58,59^ In this study, we observed the aberrant expression of the RNA m6A methyltransferase METTL14, indicating that RNA m6A modification is involved in steatosis/MASH-related inflammatory-cancer transformation. This study explored the molecular events related to the low expression of METTL14 protein during the evolution of steatosis-MASH-liver fibrosis, including the m6A modification of *GLS2* mRNA, the METTL14/GLS2/oxidative stress microenvironment signal axis, and the regulatory mechanism of macrophages and HSCs related to the progression of steatosis-MASH-hepatic fibrosis, which will help to improve the understanding of the essence of inflammatory-cancer transformation and provide theoretical and technical support for the specific prevention and treatment of MAFLD-related HCC. Oxidation and antioxidation are relatively balanced in the normal human body, but due to insulin resistance and excessive mobilization of peripheral fat in MAFLD patients, too many free fatty acids will be produced, resulting in β oxidation overload.^60^ Due to excessive oxidation, the antioxidant capacity of impaired hepatocytes decreases, requiring more antioxidant factors to resist oxidation. With the gradual progression of MAFLD, the dynamic equilibrium between oxides and antioxidants becomes unbalanced, resulting in lipid peroxidation and DAMPs release from impaired hepatocytes.^61^ Integrating the proteomic results of liver tissue of HFD-fed mice and *Mettl14* hepatocyte-specific knockout mice, it was found that the expression of glutathione-related enzymes GLS2 rather than GS in liver tissue of the two experimental groups was significantly downregulated compared with their respective controls. GLS2 is a mitochondrial phosphate-activated glutaminase that catalyzes the hydrolysis of glutamine to stoichiometric amounts of glutamate and ammonia. Consistent with these functions of GLS2, downregulated METTL14 decreased the expression of GLS2 by interfering the translation process mediated by YTHDF1, consequently resulting in decreased glutamate levels and elevated ROS levels in hepatocytes.^14,15^ This process also promoted the liver oxidative stress microenvironment and induced damaged hepatocytes to release DAMPs including 8-OHdG. It was also discovered that overexpressing GLS2 in KO mice could rescue the antioxidant capacity and reduce the ROS levels, which consequently ameliorated liver injury, inflammation and fibrosis in the *Mettl14*-deficient mice. It is noteworthy that the antibody utilized in western blotting experiments to detect GLS2 exhibited a solitary band in mouse samples but multiple bands in human samples. This result aligns with prior publications, and the potential underlying factors remain unknown, which deserve our further attention.^62,63^

The results of scRNA-seq showed that the proportion of KCs decreased and the proportion of Mo-macs increased in *Mettl14* hepatocyte-specific knockout mice, which was consistent with the change in macrophage lineage in MASH liver tissue.^23^ A new class of macrophages, namely, *Cx3cr1*^+^*Ccr2*^+^ Mo-macs, might play a key role in the progression of MASH.^28^ We compared the DEGs between *Cx3cr1*^+^*Ccr2*^+^ Mo-macs and found that the expression of inflammation activation-related genes was significantly upregulated. Further analysis of the characteristic expression genes of the two subpopulations (M06 and M09) belonging to *Cx3cr1*^+^*Ccr2*^+^ Mo-macs showed that M09 exhibited a high level of expression for M1 macrophage-associated marker molecules, which resembled SAMs or LAMs that have been shown to play crucial roles in the progression of MAFLD.^2,33^ While M06 neither expressed M1 macrophage marker molecules nor M2 macrophage cell marker molecules but highly expressed *S100a4*, *S100a6*, *S100a10* and *S100a11*. In a previous study, researchers found that a class of macrophages secreting S100A4 could activate HSCs and promote liver fibrosis.^35^ In addition, a study showed that S100A11 could activate HSCs and liver fibrosis via the regulation of the deacetylation of Smad3 in the TGF-β pathway.^64^ Based on our results and these reports, we believe that *Cx3cr1*^+^*Ccr2*^+^ Mo-macs with high expression of S100A family genes may play an important role in the progression of MAFLD-related inflammation and liver fibrosis.

During the tissue damage process including MAFLD progression, it is known that damaged hepatocytes release DAMPs, which are recognized by PRRs and then recruit immune cells to initiate an inflammatory response in the oxidative stress microenvironment.^38–41^ Since TLRs are prominent receptors for released DAMPs, we found that several TLRs were only expressed in M09 clusters, while the adaptor protein *Myd88* was expressed in both M06 and M09 clusters of *Cx3cr1*^+^*Ccr2*^+^ Mo-macs. In the present study, we discovered that CX3CR1 could interact with MyD88 and subsequently influence the downstream signaling pathways such as NF-κB, which regulated the transcription of S100A4 in *Cx3cr1*^+^*Ccr2*^+^ Mo-macs; On the contrary, extracellular S100A4 has been reported to regulate the activation of NF-κB signaling pathway^49,50^ and a mutually reinforcing regulatory interaction between them could collectively enhance the course of inflammation. Interfering the CX3CR1/MyD88/NF-κB signaling pathway using the inhibitor or activator could regulate the expression of S100A4 in PMA-treated THP-1 cells with or without high-fat treatment. Mo-macs could also stimulate S100A4 expression of HSCs under high-fat conditions. Furthermore, an in vivo experiment displayed that inhibiting MyD88 with ST2825 could alleviate liver injury, inflammation and fibrosis in *Mettl14*-deficient mice.

In conclusion, our study demonstrates that depletion of hepatocyte-specific METTL14 or MAFLD-induced downregulation of METTL14 contributes to decreased levels of GLS2 in an m6A-dependent manner, and substantiates their crucial roles in the progression of MAFLD. Our study also reveals the *Cx3cr1*^+^*Ccr2*^+^ Mo-macs that specifically drive the progression of MAFLD via CX3CR1/MyD88/NF-κB/S100A4 signaling pathway. More importantly, restoration of METTL14 or GLS2, or inhibition of MyD88 could alleviate liver injury and fibrosis, hence providing a rational basis for the development of new treatment strategies for MAFLD.

## Materials and methods

### Human samples

Five MAFLD and control liver tissues were the para-tumor tissues obtained from patients at the time of hemangioma surgery. These patients were confirmed with no history of liver disease of other etiologies including viral hepatitis, alcohol and drug use. Qualified pathologists performed histological assessments of MAFLD and determined the MASH activity score (MAS). MAFLD, or MAFL, was diagnosed when the frequency of steatosis hepatocytes exceeded 5%.^65^ Liver tissue with MAS greater than 4 was diagnosed as MASH. Four-point scoring was used to determine the level of fibrosis: 1 represented mild to severe zone 3 perisinusoidal fibrosis, or portal fibrosis alone; 2 represented zone 3 and portal/periportal fibrosis; 3 represented bridging fibrosis; and 4 represented cirrhosis.^66^ HBV-associated liver tissues were also the para-tumor tissues obtained from HCC patients who underwent hepatectomy. All subjects provided written informed consent prior to sample collection, which was approved by the Ethics Committee of Eastern Hepatobiliary Surgical Hospital (EHBH). We do not have information available about the gender of the subjects.

### Statistical analysis

Statistical analysis was performed using GraphPad Prism 9 software and R software 4.0.3. All results are presented as means ± standard error of the mean (SEM). Student’s t test and ANOVA analysis were performed for the comparison of two groups or multiple groups with a normal distribution. The m6A binding sites were predicted using the SRAMP database.^18^ The promoter binding sites of transcription factors were predicted using the JASPER database, the hTFtarget database, and the PROMO database.^51–53^ Statistical significance was indicated by *p* value (*p* < 0.05). *p < 0.05, **p < 0.01, ***p < 0.001, ****p < 0.0001.

The remaining materials and methods are described in Supplementary Data.

### Supplementary information

Supplementary data

Supplementary Table S1

Supplementary Table S2

Supplementary Table S3

## Data Availability

The scRNA-sequencing data are available in figshare with the identifier (10.6084/m9.figshare.25393228). The original proteome sequencing and metabonomic analysis in this article have been deposited in this article. All data supporting the findings of this study are available from the corresponding author upon reasonable request.
