# Supplementary material for: METTL14 downregulation drives S100A4+ monocyte-derived macrophages via MyD88/NF-κB pathway to promote MAFLD progression
Source: Signal Transduct Target Ther. 2024 Apr 17;9:91. doi: 10.1038/s41392-024-01797-1 (PMC11021505; doi:10.1038/s41392-024-01797-1)
Supplement: Supplementary file 1 — Supplementary data [file 41392_2024_1797_MOESM1_ESM.docx]

Supplementary Materials for

METTL14 downregulation drives S100A4^+^ monocyte-derived macrophages via MyD88/NF-κB pathway to promote MAFLD progression

Yue-fan Wang^1,2*^, Wen-li Zhang^2*^, Zhi-xuan Li^3*^, Yue Liu^4*^, Jian Tan^1*^, Hao-zan Yin^1^, Zhi-chao Zhang^2^, Xian-jie Piao^2^, Min-hao Ruan^2^, Zhi-hui Dai^1^, Si-jie Wang^1^, Chen-yang Mu^1^, Ji-hang Yuan^1^, Shu-han Sun^1^, Hui Liu^2†^ and Fu Yang^1, 5, 6†^

^*^These authors contributed equally.

^†^Corresponding author: Fu Yang, email: [yangfusq1997@smmu.edu.cn](mailto:yangfusq1997@smmu.edu.cn), The Department of Medical Genetics, Naval Medical University, Shanghai, 200433, China. Hui Liu, email: [liuhuigg@hotmail.com](mailto:liuhuigg@hotmail.com), The Third Department of Hepatic Surgery, Eastern Hepatobiliary Surgery Hospital Affiliated to Naval Medical University, Shanghai, 200438, China.

**This file includes:**

Materials and Methods

Supplementary Figures. S1 to S12

Supplementary Tables. S4 to S6

**Materials and Methods**

**Animal models**

**High-fat diet mouse model**

Male C57BL/6J mice, 6 weeks old, were fed a high-fat diet (HFD) containing 60 kcal% fat, 20 kcal% protein, and 20 kcal% carbohydrate, which was purchased from Dyets (cat# HF60). For the conventional HFD mouse model, mice were fed a HFD or a control diet for 24 weeks and were then sacrificed for further studies (Fig. 1c). All mice were maintained under pathogen-free conditions at room temperature with a 12-hour light/dark cycle. All animal protocols were approved by the Institutional Animal Care and Use Committee at Naval Medical University (Shanghai, China).

**Western diet mouse model**

Male C57BL/6J mice, 6 weeks old, were fed a control diet or Western diet (WD) with 41% sucrose and 1.25% cholesterol, which consisted of 40 kcal% fat, 17 kcal% protein, and 43 kcal% carbohydrate and was purchased from Dyets (cat# D18061501). Twenty weeks later, the mice were sacrificed, and the livers were resected for further studies (Supplementary Fig. 1f).

**AMLN diet mouse model**

Male C57BL/6J mice, 6 weeks old, were fed a control diet or Amylin liver NASH diet (AMLN), which consisted of 40 kcal% fat, 22% fructose and 2% cholesterol and was purchased from Dyets (cat# AMLN). Twenty-eight weeks later, the mice were sacrificed, and the livers were resected for further studies (Supplementary Fig. 1i).

**Adeno-associated virus (AAV)-treated mouse model**

After validating the efficiency of the plasmid vectors overexpressing METTL14 and GLS2, we constructed AAVs overexpressing *Mettl14* and *Gls2* named GPAAV-CMV-Mouse_*Mettl14*/*Gls2*-EF1-ZsGreen1-WPRE. For HFD mice with AAV treatment, KO and WT mice at 6 weeks old were treated with HFD. Four weeks later, Mettl14-overexpressing AAVs or control vectors were treated with caudal vein injection. In detail, KO mice were randomly assigned to the OV-AAV or NC-AAV group, while WT mice were consistently injected with NC-AAV. All mice were then fed a HFD for another 12 weeks.

Overexpressing *Gls2* AAVs were utilized for WT mice with WD/CCl_4_ treatment and KO mice. Six-week-old male C57BL/6J mice were fed a Western diet and treated with intraperitoneal injections of CCl_4_ to develop NASH. Four weeks later, *Gls2*-overexpressing AAVs or control vectors were randomly injected via the caudal vein. Then, all the mice were treated with WD and CCl_4_ injection for another 12 weeks (Fig. 8). Moreover, six-week-old *Mettl14* KO mice were fed a control diet and randomly injected with *Gls2*-overexpressing AAVs or control vectors through the caudal vein. Then, the mice were sacrificed eight weeks later (Supplementary Fig. 4).

**MyD88 inhibitor ST2825-treated mouse model**

ST2825 is a MyD88 selective inhibitor that interferes with MyD88 homodimerization, which could block IL-1β, TLR, and NF-κB signaling. It was purchased from MedChemExpress (cat# HY50937). Six-week-old *Mettl14* KO mice were fed a control diet and randomly treated with intraperitoneal injections of ST2825 at 10 mg/kg or coil oil once a week. Eight weeks later, the mice were sacrificed. Blood samples, tissue samples and photographs of them were collected for further studies (Supplementary Fig. 10).

**Mouse knockout and genotyping strategy**

As shown in Supplementary Fig. 2a, the *Mettl14* conditional knockout mouse model (C57BL/6J) was created by CRISPR/Cas-mediated genome engineering. Briefly, the Mettl14 gene is located on mouse chromosome 3, which includes 11 exons, with the ATG start codon in exon 1 and the TAG stop codon in exon 11. We selected exon 2 as a conditional knockout region (cKO region), whose deletion should result in frameshift and loss of function of Mettl14. To engineer the targeting vector, homologous arms and a cKO region were generated by PCR using BAC clone RP24-338F17 as a template. Cas9, gRNA and the targeting vector were coinjected into fertilized eggs for cKO mouse production. The pups were then genotyped by PCR, followed by sequencing analysis. The primers for the constitutive KO allele were as follows: F: 5’-GGTGAACCTGATGAGCATTTCATT-3’; R: 5’-GCTGCAGTATTAGGTACAAACACG-3’ (wild-type allele: 201 bp/conditional KO allele: 251 bp). The primers for the Albumin Cre allele were as follows: F: 5’-GAAGCAGAAGCTTAGGAAGATGG-3’; R: 5’-TTGGCCCCTTACCATAACTG-3’ (Cre allele: 390 bp).

**Cell lines**

HUH7, Hepa1-6, 293T and LX2 cells were cultured in DMEM (Gibco, USA), while Hep3B cells were cultured in MEM (Gibco, USA), and THP-1 cells were cultured in RPMI 1640 medium (Gibco, USA) with 0.05 mM β-mercaptoethanol. The cultured medium contained 10% FBS (Gibco, USA), 1% penicillin, streptomycin (Invitrogen, USA) and anti-prophylactic treatment (InvivoGen, USA) in a 5% CO2 incubator at 37 ℃. THP-1 cells were treated with 100 ng/ml phorbol myristate acetate (PMA) to construct a macrophage model in vitro.

**Lentivirus and plasmid construction and infection/transfection**

We constructed an overexpressed METTL14 lentivirus named pRLenti-EF1-EGFP-P2A-Puro-CMV-METTL14-3xFLAG-WPRE and an interfering METTL14 lentivirus named pRLenti-CMV-EGFP-2A-Puro-U6-shRNA (METTL14). Furthermore, we constructed an overexpressed YTHDF1 lentivirus named PGMLV-CMV-H_YTHDF1-3×Flag-EF1-ZsGreen1-T2A-Puro and an interfering lentivirus named pLKD-CMV-EGFP-2A-Puro-U6-shRNA (YTHDF1). For lentiviral infection, a certain amount of viruses was directly added to HUH7 cells inoculated in 6-well plates based on the levels of multiples of infection (MOI) as well as cell types. After the cells were cultured for 48 hours, the medium was replaced, and the transfection efficiency was evaluated. Cells with lentivirus required 2-4 µg/ml puromycin treatments for at least two weeks, after which they were collected and used for further experiments.

We also constructed an overexpressed YTHDF1 plasmid named pSLenti-EF1-EGFP-P2A-Puro-CMV-YTHDF1-3×FLAG-WPRE and a corresponding mutation plasmid introducing two-point mutations K395A and Y397A in the YTH domain of YTHDF1 with a 3×FLAG tag (YTHDF1-mut) named pSLenti-EF1-EGFP-P2A-Puro-CMV-YTHDF1 (K395A, Y397A)-3×FLAG-WPRE, which could abolish the binding capacity of YTHDF1 with mRNA.^1^ We also generated an overexpressed mouse *Gls2* plasmid for further AAV preparation. To explore the m6A-dependent regulation of METTL14 on *GLS2* mRNA, we generated three plasmids expressing fragments of human GLS2 and corresponding plasmids with mutations in m6A binding sites. The wild type or mutant-1/-2/-3 of GLS2 was inserted behind the F-luc coding region and was named PGL3-CMV-LUC-H_GLS2 WT1/2/3 and MT1/2/3, respectively. To evaluate the potential binding capacity of RELA (p65) on the promoters of S100A4, we first predicted the binding sites of RELA on the promoters via online databases (JASPER, hTFtarget, and PROMO). Based on comprehensive evaluations of the rating scores and possibility among the three databases, we chose three potential binding sites and then validated the binding capacity through CUT-RUN and QRT-PCR assays. We finally determined the third site with the most enrichment and constructed the plasmid named the pGL3-basic-H_S100A4 promoter (-1946 to +54) wild-type/mutant (mutation of the third site). We also generated Renilla luciferase (R-luc), named PGMR-TK. R-luc was used to normalize firefly luciferase (F-luc) activity.

For plasmid transfection, the medium was replaced with fresh medium, and the cells were transfected using Lipofectamine 3000 reagent (Invitrogen) with vector control or plasmid construct according to the manufacturer’s instructions. Forty-eight to 72 hours later, the cells were harvested for further studies.

**Dual-luciferase reporter assay**

To evaluate the m6A-dependent effects of METTL14 on GLS2 expression, HUH7 and Hep3B cells overexpressing METTL14 or control vectors were seeded in 6-well plates at 40%-60% intensity. Then, both PGL3-CMV-LUC-H_GLS2 WT1/2/3 and MT1/2/3 and PGMR-TK were transfected into oe-METTL14 and ctl-METTL14 cells. Forty-eight hours later, the cells were harvested for luciferase detection.

To explore the potential m6A-dependent regulation of YTHDF1 on the mRNA of *GLS2*, both PGL3-CMV-LUC-H_GLS2 WT1/2/3 and MT1/2/3 and PGMR-TK were transfected into oe-YTHDF1 and ctl-YTHDF1 cells for 48 hours. Then, the cells were collected for further experiments. Furthermore, both PGL3-CMV-LUC-H_GLS2 WT1/2/3 and oe-YTHDF1 wt/(K395A, Y397A) mut/vector were in permutations and combinations and were also co-transfected with PGMR-TK into HUH7 and Hep3B cells for 48 hours. Subsequently, the cells were collected for further luciferase assays.

The promoter activity of S100A4 was also measured by a dual-luciferase reporter assay. Briefly, 293T cells were transfected with the pGL3-basic-H_S100A4 promoter (-1946 to +54) WT/MT and PGMR-TK for 48 hours. Then, the cells were harvested for further studies. Firefly luciferase (F-luc) and Renilla luciferase (R-luc) were assayed by the Dual-Glo Luciferase Assay System (Promega, cat# E1910). Renilla luciferase (R-luc) was used to normalize firefly luciferase (F-luc) activity. Relative fluorescence activity was the ratio of F-luc to R-luc activity. Moreover, the procedures followed the manufacturer’s instructions and were finally detected by Spectral Max (BioTek Synergy 2, USA). Experiments were performed three times with similar results.

**High-fat cell model and bioactive molecule treatment**

To construct a high-fat cell model, HUH7 and Hep3B cells were treated with 12 μmol/l oleic acid and 6 μmol/l palmitic acid for 48 hours. Oil red O and ROS detection were used to verify the efficiency of high-fat stimulation. Subsequently, the centrifuged supernatants were added to PMA-treated THP-1 cells as conditioned medium (CM) for 24 hours. In our studies, we used several bioactive molecules, such as the MyD88 inhibitor ST2825 at 10 μM (MCE, cat# HY-50937), the CX3CR1 inhibitor JMS 17-2 at 5 μM (MCE, cat# HY-123918), the NF-κB inhibitor JSH-23 at 30 μM (MCE, cat# HY-13982), and the NF-κB activator diprovocim at 5 nM (MCE, cat# HY-123942). These bioactive molecules were added to PMA-THP-1 cells with/without CM stimulation for 24 hours to explore the upstream regulation of S100A4. For cocultivation experiment, 2×10^6 LX2 cells were cultured with the supernatants centrifugated from high-fat cells in the six-well plates and were either co-cultured or not with THP-1 induced monocyte-derived macrophages *in vitro* for 24 hours. Then LX2 cells were collected for detections of HSC activation markers.

**Cleavage under target and release using nuclease (CUT&RUN) assay**

The interaction between RELA (p65) and the promoters of S100A4 was measured using the CUN&RUN Assay Kit (CST, cat# 86652) according to the manufacturer’s instructions. Briefly, PMA-treated THP-1 cells were fixed on concanavalin A magnetic beads, the cell membranes were permeated with digitonin, and then anti-p65 and protein AG-MNase were added. The anti-p65 antibody recruited Protein AG-MNase to the target protein of chromatin, then activated MNase with an appropriate concentration of Ca^2+^, and used the enzyme cleavage property of MNase to cut DNA on both sides of the target protein and release the cut DNA fragments. Subsequently, the DNA fragments were purified and enriched, which can also be detected and quantified by QRT-PCR using the primers of S100A4 in Table S5.

**RNA and protein stability/degradation assay**

To evaluate RNA stability, cells were seeded in 6-well plates at 80% intensity and incubated with actinomycin D (Abcam #ab141058) at 5 μg/ml. Cells were collected at the indicated times, and RNA isolation was performed. The degradation rate and half-life of *GLS2* mRNA were analyzed by qRT-PCR, which was normalized to β-actin as described previously.

To evaluate protein stability, cells were treated with 100 μg/ml cycloheximide (CHX) for the indicated times and harvested. Furthermore, cells were treated with the proteasome inhibitor MG132 at 1 μM for 24 hours and harvested. Then, the protein expression of GLS2 was determined by western blot analysis.

**Coimmunoprecipitation (Co-IP) assay**

A co-IP assay was performed using the Immunoprecipitation Kit with Protein A+G Magnetic Beads (Beyotime, cat# P2179M) as described in the manufacturer’s instructions. Briefly, PMA-treated THP-1 cells were treated with lysis buffer. After centrifugation, the supernatant was collected and incubated with protein A/G magnetic beads and anti-CX3CR1 or anti-MyD88 for 24 hours at 4 °C. The precipitates were then washed three times with lysis buffer and boiled with 2× loading buffer for further Western blot analysis.

**Serum biochemistry detection**

Several kits were used to detect the serum levels of alanine aminotransferase (ALT, cat# S03030), aspartate aminotransferase (AST, cat# S03040, Rayto), total bilirubin (TBIL, cat# C120, Rayto), triglycerides (TG, cat# S03027, Rayto) and cholesterol (CHO, cat# S03042, Rayto). Then, the samples were analyzed using an automated chemical analyzer (Chemray 240/420/800, Rayto, China).

**ELISA assay**

Serum cytokines HGF, FGF2, MMP2, S100A4, and 8-OHdG in mice were detected with ELISA kits (MIBIO cat# ml037987/037702/026255/037783/002198) according to the manufacturer’s instructions.

**Nucleic acid isolation, reverse transcription PCR and real-time quantitative PCR**

All the reagent supplies are treated with RNase removal. Total RNA was isolated from cells and tissues by TRIzol reagents (Thermo Fisher, USA) or an RNA isolation kit (Vazyme#R701, Dalian, China). The concentration and quality of RNA were assessed by Nandrop 2000 (Thermo Fisher). RNA was stored at -80 ℃ until use, and complementary DNA was synthesized with reverse transcription reagents (Takara#RR036A) according to the manufacturer’s instructions. Quantitative real-time PCR (qRT-PCR) was used to detect the relative transcription expression of target genes using ChamQ Universal SYBR qPCR Master Mix (Vazyme#Q771-02). The samples were normalized to the expression of 18S (human) or ACTB (mouse) using the 2^-∆∆CT^ method. The sequences of the primers are shown in Supplementary Table 5.

**Protein extraction and western blotting (WB)**

Briefly, proteins were extracted from fresh frozen tissues or cultured cells with RIPA lysis buffer and 1 mM PMSF (Beyotime, China) according to the manufacturer’s instructions. All procedures were performed on ice. Then, the lysates were separated by 10% SDS-polyacrylamide gel electrophoresis and transferred to nitrocellulose (NC)
membranes. The membranes were immersed in quick block buffer (Epizyme, China) and then probed overnight at 4°C in solutions with primary antibodies. Next, the membranes were incubated with fluorescent secondary antibodies after the wash-up of
primary antibodies. Finally, protein levels were detected and evaluated by an Odyssey infrared fluorescent scanner (LICOR, America). The relative expression levels were normalized to the endogenous control (β-actin) and quantified based on gray values. The details of the antibodies are shown in Supplementary Table 6.

**Immunohistochemistry and immunofluorescence**

For immunohistochemistry (IHC) assays, tissue sections were dewaxed, subjected to antigen retrieval, blocked with 10% goat serum, and then incubated with primary antibodies overnight at 4 °C as well as secondary antibodies at 37 °C for 60 minutes. Nuclei were stained with DAB, hematoxylin, and hydrochloric acid. Finally, the sections were scanned or observed under a microscope. For immunofluorescence (IF) assays with two targets, the procedures before incubation with secondary antibodies were similar to IHC. Taking F4/80^+^S100A4^+^ double-positive staining as an example, tissue sections were incubated with F4/80 and goat anti-rabbit IgG (HRP). After CY3 staining, antigen retrieval, and blocking, the primary antibody was added. Then, tissue sections were incubated with S100A4 and Alexa Fluor®647 goat anti-rabbit lgG (H+L). For immunofluorescence (IF) assays with three targets, such as CX3CR1^+^CCR2^+^MYD88^+^ staining, we used a tyramide signal amplification (TSA) method, which is a high-density in situ labeling method for the detection of target proteins based on the catalytic activity of horseradish peroxidase HRP. We added three different primary antibodies in sequence and performed elution, incubation of secondary antibodies and staining. DAPI and anti-fluorescence quenching agents were used to stain nuclei and for storage. Tissue sections were scanned by 3DHistech (Pannoramic MIDI, Hungary). The details of the antibodies are listed in Table S6. Relative quantification of targets in IHC and IF assays depended on the levels of average optical density (AOD) evaluated by Image Pro Plus 6.0 software (Media Cybernetics, USA).

**Single-cell RNA sequencing**

We used the Countess II Automated Cell Counter (Thermo Fisher Scientific, USA) to count cells waiting to be tested and adjusted the concentration to an ideal concentration of 1 × ${10}^{6}$/ml. Then, cDNA was marked by 10× GemCode Technology. Gel beads containing barcode information were first mixed with cells and enzymes. Droplets flowed into the reservoir, were collected, and then dissolved and released primer sequences for reverse transcription. cDNA was used as a template to amplify PCR. A sequencing library was constructed by mixing products containing barcode amplification information in each droplet. First, DNA fragments were broken into 200-300 base pair fragments by a Biorupter Ultrasound Fragmentation Instrument. Next, the DNA library was amplified by PCR with sequencing connector P5 and sequencing primer R1. Finally, prepared samples were subjected to the 10× single-cell sequencing analysis platform.

**Data processing and quality control**

Analyses were performed by using R software version 4.0.3 and the "Seurat" package version 3.1.1. Cells with detectable genes below 200 and mitochondrial content greater than 10% were filtered out during the quality control process.

**Dimensionality reduction analysis**

After excluding low-quality cells, we then used the ‘NormalizeData’ function to normalize filtered UMI counts, for which the normalization method was set as ‘logNormalize’, with a scaling factor of 10000. The ‘FindVariableGenes’ function was used with default parameters to select highly variable genes, which were subsequently applied for linear dimensionality reduction. Next, principal component analysis (PCA) was performed using the top 2000 highly variable genes via the ‘RunPCA’ function. Thereafter, a number of PCs were selected corresponding to ElbowPlot, followed by performing the ‘RunUMAP’ function with a perplexity value of 30 to acquire bidimensional coordinates for a single cell. Meanwhile, we used unsupervised cell clustering by the ‘FindClusters’ function with a resolution of 0.6 based on identical PCs as for the ‘RunUMAP’ function. Consequently, datasets were visualized by UMAP plots.

**Differentially expressed gene analysis**

Based on normalized data, the functions "FinAllMarkers" or "FindMarkers" were used to identify DEGs across different clusters. We applied the Bonferroni correction method to adjust the *P* value and excluded DEGs whose adjusted P value was higher than 0.05. A nonparametric Wilcoxon rank sum test from the "Seurat" package was designated for differential expression analysis between each subpopulation.

**Trajectory and pseudotime analysis**

"Monocle2" software was used to identify differential genes that changed between clusters or spots for developmental trajectory and pseudotime analysis. The "Monocle2" package for trajectory and pseudotime analysis contained 400 marker genes from the "differentialGeneTest" function that is designed to infer potential pedigree differentiation trajectories. A generalized additive model (GAM) is constructed to generate the average expression of each isotype. RNA counts of all spots in the cluster were selected as the input of "Monocle2" for downstream analysis.

**Proteomic analysis**

The proteomic analysis of liver tissues from the Mettl14 KO (n=6) and WT (n=4) mice, as well as HFD-fed (n=3) & control diet (n=3) was performed. The samples were treated with 300 µL of lysis buffer and 1 mM PMSF and further lysed with sonication. BCA assays were used to detect the protein concentration, and the samples were then stored at -80°C. Next, the samples were separated by a 12% SDS-PAGE gel, which was further fixed for 2 h and stained for 12 h. After staining, the gel was washed to visualize the bands. Finally, the stained gel was scanned by an automatic digital gel image analysis system. The solutions were collected and lyophilized by the filter-aided sample preparation (FASP) method. Then, the peptide samples were labeled with isobaric tags for the relative and absolute quantification (iTRAQ) method. After labeling, the samples underwent desalination and phosphopeptide enrichment for further mass spectrometry and data processing. All analyses were performed by a Q-Exactive HF mass spectrometer (Thermo, USA) equipped with a Nanospray Flex source (Thermo, USA). The original data were in supplementary datasets (Table S1-2).

**Metabonomic analysis**

We applied an ultrahigh-performance liquid chromatography mass spectrometry (UPLC-MS) system to perform the liver tissue analysis. All samples were analyzed by a reversed-phase liquid chromatography (RPLC) XSelect HSS T3 (2.1 mm × 100 mm, 2.5 µm, Waters, Milford, USA) column as previously described ^2^. The samples were randomly injected into the LC to reduce experimental drifts. In-house quality control was prepared by pooling and mixing the same volume of each sample. Further extraction of metabolites from liver tissue samples, data acquisition and processing, metabolite identification are performed. Finally, the UPLC-MS data obtained from the T3 column was integrated into a new database for metabolomic profiling.

The relative intensities of metabolites were normalized using L-2-chlorophenylalanine as the internal standard and then subjected to multivariate processing. The data were imported into SIMCA (version 14.0, Umetrics, Umeå, Sweden) to perform orthogonal partial least square discriminant analysis (OPLS-DA). Variable importance in projection (VIP) was used to measure the discriminatory power of the metabolites. The original data was depicted in Table S3.

**Cytokine protein microarray**

The protein extracts from liver tissues of KO and WT mice (n=8) were analyzed for the presence of cytokines using a Mouse Cytokine Antibody Array to screen for differential expression of 200 proteins, including cytokines, chemokines, inflammatory mediators, growth factors, and matrix metalloproteinases (H-Wayen Biotechnologies, Shanghai, China).

**M6A RNA immunoprecipitation**

As the RIP kit (Millipore, USA) instructed, liver tissues from KO, HFD, and corresponding control mice were treated with TRIzol solution for total RNA extraction. One hundred micrograms of extracted RNA from each group was prepared for further studies. Prepare a magnetic bead with an anti-m6A antibody mixture and incubate RNAs with the magnetic bead antibody mixture. Perform RNA purification and qRT-PCR analysis of *GLS2* mRNA. Then, the result was verified by 2% agarose gel electrophoresis. For relative quantification analysis, the ∆CT value was calculated according to the CT value of the target group, the IgG group as the CT value of the input group was the reference, and 2^-∆CT^ was used for relative quantification.

**Liver histology and staining**

We performed several staining methods to acquire liver histological assessment and observe the degrees of collagen and lipid deposition in the liver sections, including hematoxylin-eosin (HE) staining, Sirius red staining, Masson staining, and oil red O staining.

**HE staining**

Briefly, paraffin sections were dewaxed in water via gradient alcohol, and then hematoxylin was used to stain the nucleus, and eosin was used to stain the cytoplasm. The sections were successively put into gradient alcohol for dehydration and xylene for transparency. Then, the sections were removed from the xylene, slightly dried, and sealed with neutral gum. Finally, microscopic observation, image acquisition, and analysis were performed. For image evaluation, the entire section was scored semi-quantitatively for three different categories of pathology: steatosis, inflammation, and liver cell injury, which were reported as the MAFLD Activity Score (MAS).^3^

**Sirius red staining**

For Sirius red staining, the tissue was fixed with a neutral formaldehyde solution, paraffin sectioned, and routinely dewaxed with water. Then, the tissue was dyed with celestine blue solution for 5-10 minutes and washed with distilled water. Then, the tissue was dyed with Sirius red picric acid for 15-30 minutes. Sections were then dehydrated with absolute ethanol, cleared with xylene, and sealed with neutral resins.

**Masson staining**

For Masson staining, the dehydrated sections were soaked in Bouin's solution and rinsed well with running water. The sections were stained with hematoxylin for 5-10 minutes and then washed slightly with running water. The sections were treated with 1% hydrochloric acid alcohol, rinsed with water for several minutes, returned blue with lithium carbonate for a few seconds and rinsed with water. The sections were stained with Li Chunhong acid fuchsin solution for 5-10 minutes, rinsed slightly with running water, and treated with phosphomolybdic acid solution for 5 minutes. After that, the cells were stained directly with an aniline blue solution for 5 minutes without washing. The sections were treated with 1% glacial acetic acid for 1 minute and dehydrated with 95% alcohol many times. Then, sections were treated as previously described.

**Oil Red O staining**

For oil red O staining, the fixed sections or cultured cells were rinsed in 60% isopropyl alcohol for 20-30 seconds. Six milliliters of oil red O storage solution was added to 4 ml of distilled water and mixed for approximately 10 minutes for further dyeing. Then, the sections were placed in oil red O staining solution for 5-10 minutes. Remove the excess dye slightly with 60% isopropyl alcohol and wash with distilled water. After nuclear staining, the cells were rinsed with water for 10 minutes or returned blue to a dilute lithium carbonate aqueous solution and washed with water. The water around the slice was wiped with filter paper, and then the sections were treated as previously described.

**Primary hepatocyte isolation**

All procedures were performed under sterile conditions. After the mice were anesthetized properly based on body weight, the abdomen was exposed, the inferior vena cava and portal vein were found, and the retention needle was inserted into the portal vein. The liver was perfused slowly with calcium-free Hank’s balanced salt solution (HBSS) for approximately 10-20 minutes to remove the blood of the mice. When it was finished, perfusion was continued with switching to a 1.6 mg/ml collagen IV solution (Worthington, LS004188). After rinsing, the mouse liver was cut off and transferred to DMEM high glucose medium 2-3 times. Tear the liver capsule in the medium using sterilizing tweezers to allow the primary cells to flow out of the liver. The prepared single-cell suspension was centrifuged twice at 50 g with an 80% brake at 4°C for 3 min, the supernatant was then restored to isolate non-parenchymal cells, and the parenchymal cells were resuspended by precipitation of 1~2 ml medium. The parenchymal cell suspension was once again added to 100% Percoll (2:1) and centrifuged at 70 g for 10 minutes at 4 ℃, and the precipitate was retained. Cell viability was counted and calculated, and if the viability was higher than 80%, the isolated primary cells were eligible for further sequencing studies and intracellular ROS level evaluation. Nonparenchymal cells were added to 100% Ficoll (2:1) and centrifuged at 800 g and 4 ℃ for 20 minutes. The cells of the interface layer were carefully transferred to another centrifuge tube, diluted with 10 ml of HBSS solution, and centrifuged at 1500 rpm/min at 4 ℃ for 5 minutes to precipitate the cells. The collected nonparenchymal cells were then centrifuged at 650 g for 7 minutes at 4 ℃ to retain the precipitates. The following procedures were consistent with those described above for parenchymal cells.

**Measurement of intracellular and tissue ROS levels**

The intracellular ROS levels were measured using a reactive oxygen species detection kit (Beyotime Biotechnology, China) according to the operating instructions provided by the manufacturer. Briefly, the primary cultured hepatocytes were seeded in 6-well plates as described above, and then the cells were incubated with 2’,7’-dichlorofluorescein-diacetate (DCFH-DA) for 20 min at 37°C. The cells were washed three times for 5 minutes each time and stained with DAPI for 10 minutes for further observation. For tissue ROS detection, frozen tissue sections were thawed and marked on slides. Then, ROS reagents were added, and the sections were incubated at 37 ℃ for 30 minutes. The slides were placed in PBS (pH 7.4) and washed three times away from light for 5 minutes each time. After slightly drying the sections, DAPI dye solution was added, and the nuclei were stained at room temperature for 10 minutes. Then, the slides or cell plates were sealed and observed under a fluorescence microscope (Zeiss), and images were collected.

**Total antioxidant capacity (TAC) assay**

The TAC of liver tissues was measured using a TAC assay kit (Beyotime Biotechnology, China) according to the operating instructions provided by the manufacturer. The collected liver tissue was homogenized at 4 ℃ with the lysis buffer provided by the kit. The lysate was centrifuged at 12000 rpm at 4 °C for 10 minutes, and the supernatant was taken for detection. The concentration of protein was determined by the BCA method (Beyotime Biotechnology, China). Ten microliters of pyrolysis solution were mixed with 200 μl of ABTS working solution in a 96-well plate and incubated at room temperature for 5 minutes. Using a microplate reader (BioTek Instruments, Vermont, USA), the absorbance at 734 nm was measured. The TAC of each liver tissue sample was calculated according to the operating instructions provided by the manufacturer. Each measurement was performed in triplicate.

**Supplementary Figures and Figure Legends**

**Figure S1. METTL14 was downregulated in human non-alcoholic fatty liver tissue and liver tissue of fatty liver mouse models.**

(a) The number of male patients with no steatosis and minimal, mild, moderate, and severe steatosis among the high expression or low expression of METTL14 group from the GTEx liver data (n=161). (b) The *METTL14* levels of groups of patients ranging from no steatosis, minimal, mild, moderate to severe steatosis from the GTEx liver data (n=86, 18, 27 and 30 respectively). (c) The *METTL14* levels of patients with low or high severity of steatosis from the GTEx liver data. (d) Representative pathological findings in human liver tissues in terms of METTL14 expression levels from the GTEx liver data (scar bar = 50 μm). (e) Immunofluorescence staining of METTL14 and CK18 of the liver sections among high fat diet (HFD) and control diet (CON) groups (n=3, scar bar = 10 μm). METTL14 is marked in green, CK18 is marked in red and DAPI is marked in blue. (f) Schematic diagram of the dietary feeding scheme. Six-week-old C57BL/6 wild-type mice were fed either a control diet (CON) or a Western diet (HFD) for 24 weeks (n=5). (g) HE, Oil Red O and Sirius Red staining of WD-fed and control diet-fed mice (scar bar = 20 μm). (h) Western blot (left) and quantitative analysis (right) showing the expression of METTL14 and GLS2 in WD-fed and control diet-fed mice (n=5). (i) Schematic diagram of the dietary feeding scheme. Six-week-old C57BL/6 wild-type mice were fed either a control diet (CON) or an Amylin liver NASH diet (AMLN) for 28 weeks (n=3). (j) HE, Oil Red O and Sirius Red staining of AMLN-fed and control diet-fed mice (scar bar = 50 μm). (k) Western blot (left) and quantitative analysis (right) showing the expression of METTL14 and GLS2 in AMLN-fed and control diet-fed mice (n=3). Data are represented as mean ± SEM. NS not significant, *p <0.05, **p <0.01. The mouse image was created with BioRender.com.

**Figure S2. Strategy and phenotyping for Mettl14^loxp^-Alb^Cre^ conditional knockout mice.**

(a) Targeting strategy for conditional knockout mice. (b) Genotyping and identification strategy for the Mettl14-loxp and Alb-Cre alleles. (c) The mRNA levels of Mettl14 in the livers of KO and WT mice (n=4). (d) Representative image showing the protein levels of METTL14 in the livers of KO and WT mice (n=4). (e) Relative quantitative analysis of METTL14 expression in KO and WT mice (n=4). (f) Representative image showing the protein levels of METTL14 in primary cultured hepatocytes isolated from mouse livers in the KO and WT groups (n=4). (g-h) Comparison of body weight (g) and liver weight (h) of WT (n=10), KO^+/-^ (n=7), and KO (n=7) mice. (i) HE staining of liver tissue sections from KO and WT mice. (j) MASH activity score (MAS) of WT-NC (n=4), KO^+/-^-NC (n=4), KO-NC (n=5), and KO-OV (n=5) mice with steatosis, inflammation, and hepatocyte injury (ballooning). (k) Relative quantitative analysis of Oil Red O staining in WT-NC (n=4), KO^+/-^-NC (n=4), KO-NC (n=5), and KO-OV (n=5) mice. Data are represented as mean ± SEM. NS not significant, *p <0.05, **p <0.01, *** p <0.001, ****p <0.0001. The mouse image was created with BioRender.com.

**Figure S3. The expression of glutamine synthetase (GS) did not change significantly in the absence of METTL14 or MAFLD progression.**

(a-b) The protein levels of GS in the livers of control diet and HFD fed mice (a) as well as WT and KO mice (b) using the proteomic analysis (n=3). (c) The *GS* levels of groups of patients ranging from no steatosis, minimal, mild, moderate to severe steatosis from the GTEx liver data (n=86, 18, 27 and 30 respectively). (d) The *GS* levels of male patients with low or high severity of steatosis from the GTEx liver data. Data are represented as mean ± SEM. NS not significant, **p <0.01, ****p <0.0001. The mouse image was created with BioRender.com.

**Figure S4. Restoration of GLS2 in KO mice ameliorates ROS levels, liver injury, inflammation and fibrosis.**

(a) QRT-PCR analysis indicating the expression of *Gls2* mRNA in Hepa1-6 cells transfected with the GLS2 overexpression plasmid or control vector. (b-c) Western blot (b) and relative quantitative analysis (c) showing the protein levels of GLS2 in Hepa1-6 cells transfected with the GLS2 overexpression plasmid or control vector. (d) Schematic diagram of KO mice with AAV treatment. Six-week-old KO mice were randomly injected with AAV-8 overexpressing Gls2 (KO-OE) or control vector (KO-CTL) through the caudal vein and were sacrificed eight weeks later. (e) QRT-PCR analysis showing the levels of Gls2 mRNA from KO-OE and KO-CTL mice (n=6). (f) Western blot (left) and relative quantitative analysis (right) showing the expression of GLS2 in KO-OE and KO-CTL mice (n=6). (g) Representative image showing resected livers of KO-OE and KO-CTL mice (n=8). (h) Comparison of the liver-to-body weight ratio of KO-OE and KO-CTL mice. (i) The levels of serum ALT, AST and TBIL in KO-OE and KO-CTL mice (n=8). (j) Representative image showing ROS levels in KO-OE and KO-CTL mice (n=8, scar bar = 50 μm). (k) Total antioxidant capacity (TAC) analysis of OE-AAV and CTL-AAV mice (n=8, scar bar = 50 μm). (l) QRT-PCR analysis showing the expression of fibrosis markers, such as *Acta2* (n=7), *Col1a1* (n=7) and *Mmp2* (n=6)*,* in OE-AAV and CTL-AAV mice. (m) HE, Oil Red O, Sirius Red, and Masson staining and α-SMA immunofluorescence analysis of KO-OE and KO-CTL mice (scar bar = 20 μm). (n) The levels of serum S100A4 in KO-OE and KO-CTL mice (n=8). Data are represented as mean ± SEM. NS not significant, *p <0.05, **p <0.01, *** p <0.001, ****p <0.0001.

 **Figure S5. METTL14 does not affect the mRNA and protein stability of GLS2.**

(a-b) QRT-PCR analysis showing the expression of METTL14 and GLS2 in HUH7 (a) and hep3B (b) cells infected with METTL14 overexpression (OE) or shRNA lentivirus vector (SH). (c-d) Relative quantitative analysis of the protein levels of METTL14 and GLS2 in HUH7 (c) and hep3B (d) cells infected with METTL14 overexpression or shRNA lentivirus vectors. (e-f) Western blot and quantitative analysis showing the protein degradation of GLS2 in HUH7 cells infected with METTL14 overexpression lentivirus (e) or METTL14 shRNA lentivirus (f). (g) Western blot (left) and quantitative analysis (right) indicating GLS2 expression in HUH7 cells infected with METTL14 overexpression lentivirus or control vector and treated with/without MG-132 for 24 h. (h) Western blot (left) and quantitative analysis (right) indicating GLS2 expression in HUH7 cells infected with METTL14 shRNA lentivirus or control vector and treated with/without MG-132 for 24 h. (i) Model diagrams of the YTHDF1 overexpression plasmid (oe-YTHDF1-wt) and YTHDF1 mutation plasmid with K395A & Y397A sites (oe-YTHDF1-mut). (j) RIP-PCR analysis with anti-YTHDF1 antibody demonstrating YTHDF1 binding ability on GLS2 mRNA. (k) Relative quantitative analysis showing YTHDF1 and GLS2 expression in HUH7 (left) and hep3B (right) cells transfected with YTHDF1 overexpression, YTHDF1 mutation (K395A & Y397A) and vector plasmid of GLS2. (l) Relative quantitative analysis showing METTL14, YTHDF1 and GLS2 expression in HUH7 (left) cells and hep3B cells (right) infected with YTHDF1 overexpression and/or METTL14 shRNA lentivirus vector as well as control vectors. Data are represented as mean ± SEM. NS not significant, *p <0.05, **p <0.01, *** p <0.001, ****p <0.0001.

**Figure S6. The gene expression patterns and characteristics of M05 cluster.**

(a) Volcano plot comparing DEGs for the M05 cluster (|log2FC| = 0.5, p < 0.05). The top 20 genes were marked in red. (b) GO and KEGG pathway enrichment analysis of DEGs in the M05 cluster.

**Figure S7. Hepatocyte-specific knockout of *Mettl14* or HFD resulted in more CX3CR1^+^CCR2^+^ cells in the liver tissue.** (a) Representative image showing the distribution of CX3CR1^+^ and CCR2^+^ stained cells in the KO and WT groups (scar bar = 10 μm). (b) Representative image showing the distribution of CX3CR1^+^ and CCR2^+^ stained cells in the HFD and control groups. Liver tissues from mice in each group were stained with antibodies against CX3CR1 (red) and CCR2 (green). The nuclei were stained with DAPI (blue), and the arrowheads represent CX3CR1^+^CCR2^+^ cells (scar bar = 10 μm).

 **Figure S8. Developmental trajectory.** (a) The developmental trajectory of Mo-macs colored-coded by the clusters, states and pseudotime. (b) Tree diagram (left) showing the proportion of clusters in different developmental trajectory states and violin plot (right) showing the difference in the pseudotime distribution of cells between the KO and WT groups.

**Figure S9. Hepatic stellate cell activation in KO mice with hepatocyte-specific homozygous knockout of *Mettl14* in the liver microenvironment.**

(a) UMAP plot displaying the distributions of 3 hepatic stellate cell clusters, and the percentage bar plot shows the increased abundance of the HSC03 cluster in the KO group. (b) Volcano plot showing upregulated and downregulated genes in the HSC03 cluster. (c) High expression of *Col1a1*, *Col1a2*, *Col3a1* and *Col6a3* in HSC03 cells suggests functional activation of hepatic stellate cells. (d) Heatmaps showing significantly upregulated inflammation-related factors between KO and WT mice by cytokine protein microarray (n =8). (e) Heatmaps presenting the expression levels of inflammation-related factors upregulated by cytokine protein microarray across each cluster by scRNA-seq. (f-i) ELISA array detecting the serum levels of HGF (f), FGF2 (g), MMP2 (h), and S100A4 (i) from KO and WT mice (n=6). (j) Immunofluorescence analysis showing α-SMA expression in the livers of 8-week-old KO and WT mice (n=3, scar bar = 20 μm). (k) Relative quantitative analysis of α-SMA in WT and KO mice (n=3). (l) Sirius red staining showing the degree of collagen deposition and liver fibrosis in 8-week-old KO and WT mice (n=3, scar bar = 50 μm). (m) Relative quantitative analysis of collagen deposition and liver fibrosis in WT and KO mice (n=3). Data are represented as mean ± SEM. NS not significant, *p <0.05, **p <0.01.

**Figure S10. The MyD88 inhibitor ST2825 ameliorates liver fibrosis in METTL14-deficient mice.**

(a) Schematic diagram of KO mice treated with ST2825. Six- to eight-week-old KO mice were randomly intraperitoneally injected with corn oil (KO-OIL) or the myd88 inhibitor ST2825 (KO-ST2825) at 10 mg/kg once a week and were sacrificed eight weeks later. (b) Representative image of resected livers from KO-ST2825 and KO-OIL mice (n=6). (c-d) Relative body changes, liver weight and liver/body ratio of KO-ST2825 and KO-OIL mice (n=6). (e) Serum levels of ALT, AST and TBIL from KO-ST2825 and KO-OIL mice (n=6). (f) QRT-PCR analysis showing the expression of fibrosis markers, such as *Acta2*, *Col1a1* and *Mmp2,* in KO-ST2825 and KO-OIL mice (n=6). (g) Serum S100A4 levels of KO-ST2825 and KO-OIL mice. (h) HE, Oil Red O, Sirius Red, Masson staining and α-SMA immunofluorescence analysis of KO-ST2825 and KO-OIL mice (n=6, scar bar = 20 μm). Data are represented as mean ± SEM. NS not significant, *p <0.05, **p <0.01, *** p <0.001. The mouse image was created with BioRender.com.

**Figure S11.** **Restoration of METTL14 function in mice ameliorates liver fibrosis by decreasing the number of F4/80^+^S100A4^+^ cells.**

(a) Western blotting (left) and quantitative analysis (right) showing the expression of GLS2 in the CON-WT, CON-KO and HFD-KO mice with the same age (n=4). (b-c) Relative quantitative analysis of F4/80^+^ cells (b) and S100A4^+^ cells (c) among the WT-NC (n=4), KO-NC (n=5), and KO-OV (n=5) groups. (d) Relative quantitative analysis of α-SMA expression in the WT-NC (n=4), KO-NC (n=5), and KO-OV (n=5) groups. (e-f) Relative quantitative analysis of collagen deposition and fibrosis degree using Sirius Red staining (e) and Masson staining (f) in the WT-NC (n=4), KO-NC (n=5), and KO-OV (n=5) groups. (g) Immunofluorescence staining indicating the nuclear translocation and distribution of P65 in the WT-NC (n=4), KO-NC (n=5), and KO-OV (n=5) groups (scar bar = 10 μm). (h) Western blotting showing the expression of phosphorylated P65 in the WT-NC, KO-NC, and KO-OV groups (n=4). (i) Immunofluorescence staining indicating the nuclear translocation and distribution of P65 in the KO mice overexpressing *Gls2* (KO-OE) and KO-CTL groups (n=6, scar bar = 10 μm). (j) Western blotting showing the expression of phosphorylated P65 in the KO-OE and control (KO-CTL) groups (n=6). (k) Immunofluorescence staining indicating the nuclear translocation and distribution of P65 in the WD/CCl_4_ treated WT mice overexpressing *Gls2* (WD/CCl_4_-OE) and control (WD/CCl_4_-CTL) groups (scar bar = 10 μm). (l) Western blotting showing the expression of phosphorylated P65 in the WD/CCl_4_-OE and WD/CCl_4_-CTL groups (n=6). Data are represented as mean ± SEM. NS not significant, *p <0.05, **p <0.01, *** p <0.001, ****p <0.0001.

 **Figure S12. The METTL14/GLS2/MyD88/S100A4 regulation pathway was specific to MAFLD progression.**

(a) Representative images of the expression of METTL14, GLS2, MyD88, S100A4, α-SMA as well as Sirius Red staining of liver sections from two patients with low or high severity of liver fibrosis. (b-f) Quantitative analysis of the expression of METTL14 (b), GLS2 (c), MyD88 (d), S100A4 (e), α-SMA (f) among groups of patients with low severity (n=36) or high severity (n=31) of fibrosis. Data are represented as mean ± SEM. NS not significant, *p <0.05, **p <0.01, *** p <0.001, ****p <0.0001.

**Table S4. Predictions for the m6A sites of GLS2 mRNA using the SRAMP database.**

| **Number** | **Position** | **Score(combined)** | **Decision** |
| --- | --- | --- | --- |
| 1 | 292 | 0.656 | m6A site (High confidence) |
| 2 | 343 | 0.745 | m6A site (Very high confidence) |
| 3 | 350 | 0.561 | m6A site (Moderate confidence) |
| 4 | 372 | 0.642 | m6A site (High confidence) |
| 5 | 429 | 0.624 | m6A site (High confidence) |
| 6 | 750 | 0.585 | m6A site (Moderate confidence) |
| 7 | 885 | 0.657 | m6A site (High confidence) |
| 8 | 1132 | 0.538 | m6A site (Low confidence) |
| 9 | 1432 | 0.62 | m6A site (High confidence) |
| 10 | 1444 | 0.53 | m6A site (Low confidence) |
| 11 | 1458 | 0.565 | m6A site (Moderate confidence) |
| 12 | 1567 | 0.554 | m6A site (Low confidence) |
| 13 | 1572 | 0.671 | m6A site (High confidence) |
| 14 | 1655 | 0.558 | m6A site (Moderate confidence) |
| 15 | 1663 | 0.713 | m6A site (Very high confidence) |
| 16 | 1669 | 0.612 | m6A site (High confidence) |
| 17 | 1706 | 0.611 | m6A site (High confidence) |
| 18 | 1765 | 0.689 | m6A site (Very high confidence) |
| 19 | 1826 | 0.553 | m6A site (Low confidence) |
| 20 | 1846 | 0.68 | m6A site (Very high confidence) |
| 21 | 1906 | 0.662 | m6A site (High confidence) |
| 22 | 1939 | 0.613 | m6A site (High confidence) |
| 23 | 2128 | 0.585 | m6A site (Moderate confidence) |
| 24 | 2155 | 0.609 | m6A site (High confidence) |
| 25 | 2275 | 0.637 | m6A site (High confidence) |

**Table S5. Sequences of primers for quantitative real-time PCR.**

| **Genes** | **Primer sequences** |
| --- | --- |
| Hum-METTL14-F | AGTGCCGACAGCATTGGTG |
| Hum-METTL14-R | GGAGCAGAGGTATCATAGGAAGC |
| Hum-GLS2-F | GCCTGGGTGATTTGCTCTTTT |
| Hum-GLS2-R | CCTTTAGTGCAGTGGTGAACTT |
| Hum-COL1A1-F | GAGGGCCAAGACGAAGACATC |
| Hum-COL1A1-R | CAGATCACGTCATCGCACAAC |
| Hum-ACTA2-F | AAAAGACAGCTACGTGGGTGA |
| Hum-ACTA2-R | GCCATGTTCTATCGGGTACTTC |
| Hum-TGFB1-F | GGCCAGATCCTGTCCAAGC |
| Hum-TGFB1-R | GTGGGTTTCCACCATTAGCAC |
| Hum-18S-F | GGAGAGGGAGCCTGAGAAACG |
| Hum-18S-R | TTACAGGGCCTCGAAAGAGTCC |
| Hum-S100A4-F | GATGAGCAACTTGGACAGCAA |
| Hum-S100A4-R | CTGGGCTGCTTATCTGGGAAG |
| Hum-S100A4-F1(CUT-RUN) | CTTCAAGGACTGCCCCTCAG |
| Hum-S100A4-R1(CUT-RUN) | GAGACGCGTCCTCAGAGAAG |
| Hum-S100A4-F2(CUT-RUN) | GAGCTCTTTGCTCCTCTCTGG |
| Hum-S100A4-R2(CUT-RUN) | CTCAGGGTGGGCTACAAACC |
| Hum-S100A4-F3(CUT-RUN) | GACCCTGAGCCTTACTTGGC |
| Hum-S100A4-R3(CUT-RUN) | TTGCCCTGGAAATGTGGTGA |
| Mus-S100A4-F | TCCACAAATACTCAGGCAAAGAG |
| Mus-S100A4-R | GCAGCTCCCTGGTCAGTAG |
| Mus-Mettl14-F | CTGAGAGTGCGGATAGCATTG |
| Mus-Mettl14-R | GAGCAGATGTATCATAGGAAGCC |
| Mus-Actb-F | GGCTGTATTCCCCTCCATCG |
| Mus-Actb-R | CCAGTTGGTAACAATGCCATGT |
| Mus-Gls2-F | CGTCCGGTACTACCTCGGT |
| Mus-Gls2-R | TGTCCCTCTGCAATAGTGTAGAA |
| Mus-Col1a1-F | GCTCCTCTTAGGGGCCACT |
| Mus-Col1a1-R | CCACGTCTCACCATTGGGG |
| Mus-Acta2-F | GTCCCAGACATCAGGGAGTAA |
| Mus-Acta2-R | TCGGATACTTCAGCGTCAGGA |
| Mus-Mmp2-F | CAAGTTCCCCGGCGATGTC |
| Mus-Mmp2-R | TTCTGGTCAAGGTCACCTGTC |

**Table S6. Details of the antibodies used in the study.**

| **Antibody names** | **Brand** | **Product code** | **Application** |
| --- | --- | --- | --- |
| Anti-METTL14 | Sigma-Aldrich | HPA038002 | IHC, 1:1000  WB, 1:1000 |
| Anti-GLS2 | Novus | NBP1-76544 | WB, 1:1000  IHC, 1:2000 |
| Anti-GLS2 | Affinity | DF13386 | WB, 1:1000 |
| Anti-YTHDF1 | Proteintech | 17479-1-AP | WB, 1:1000 |
| Anti-F4/80 | Abcam | Ab239369 | IF, 1:100 |
| Anti-CK18 | Abcam | ab315097 | IF, 1:50 |
| Anti-m6A | SYSY | No.202003 | IP, 1:20 |
| Anti-S100A4 | Abcam | Ab197896 | IF, 1:250 |
| Anti-S100A4 | Servicebio | GB11397-80 | IHC, 1:400 |
| Anti-α-SMA | Servicebio | GB111364 | WB, 1:1000  IF, 1:500 |
| Anti-β-actin | Servicebio | GB15001 | WB, 1:1000 |
| IRdye680 Goat anti-mouse IgG | Licor | 926-32220 | WB, 1:10000 |
| IRdye800 Goat anti-Rabbit IgG | Licor | 926-32211 | WB, 1:10000 |
| Alexa Fluor®647 Goat anti-rabbit IgG (H+L) | Abcam | Ab150076 | IF, 1:400 |
| Goat anti-Rabbit IgG HRP | Abcam | Ab205718 | IHC, 1:2000  IF, 1:4000 |
| Anti-CCR2 | Novus | NBP-67700 | IF: 1:50 |
| Anti-CCR2 | Servicebio | GB11326 | IF: 1:1000 |
| Anti-CX3CR1 | Invitrogen | 14-6093-81 | WB, 1:1000  IP, 1:20  IF: 1:50 |
| Anti-MyD88 | Servicebio | GB111554 | IHC/IF, 1:1200  IP, 1:20 |
| Anti-MyD88 | Proteintech | 67919-1-Ig | IHC/IF, 1:500 |
| Anti-P65 (Ser468) | Proteintech | 82335-1-RR | WB, 1:2000 |
| Anti-IκBα | Proteintech | 10268-1-AP | WB, 1:5000 |
| Anti-p-IκBα(Ser32/36) | Proteintech | 82349-1-RR | WB, 1:5000 |
| Anti-P65 | Proteintech | 10745-1-AP | WB, 1:1000  IP, 1:20 |
| Anti-NF-κB1(p50) | Proteintech | 14220-1-AP | WB, 1:5000 |

**References**

1	Liu, T. et al. The m6A reader YTHDF1 promotes ovarian cancer progression via augmenting EIF3C translation. *Nucleic Acids Res* **48**, 3816-3831 (2020).

2	Liu, Y. et al. A distinct glycerophospholipid metabolism signature of acute graft versus host disease with predictive value. *JCI Insight* **5** (2019).

3	McGettigan, B. et al. Dietary Lipids Differentially Shape Nonalcoholic Steatohepatitis Progression and the Transcriptome of Kupffer Cells and Infiltrating Macrophages. *Hepatology* **70**, 67-83 (2019).
